# Microbial Community Profile and Water Quality in a Protected Area of the Caatinga Biome

**DOI:** 10.1371/journal.pone.0148296

**Published:** 2016-02-16

**Authors:** Fabyano Alvares Cardoso Lopes, Elisa Caldeira Pires Catão, Renata Henrique Santana, Anderson de Souza Cabral, Rodolfo Paranhos, Thiago Pessanha Rangel, Carlos Eduardo de Rezende, Robert A. Edwards, Cristiane C. Thompson, Fabiano L. Thompson, Ricardo Henrique Kruger

**Affiliations:** 1 Cellular Biology Department, Universidade de Brasília (UnB), Brasília, DF, Brazil; 2 Genomic Sciences and Biotechnology, Universidade Católica de Brasília (UCB), Brasília DF, Brazil; 3 Institute of Biology, Universidade Federal do Rio de Janeiro (UFRJ), Rio de Janeiro, Brazil; 4 Laboratory of Environmental Sciences, Universidade Estadual do Norte Fluminense (UENF), Campos dos Goytacazes, Brazil; 5 Computational Science Research Center, San Diego State University (SDSU), San Diego, California, United States of America; 6 Department of Computer Science, San Diego State University (SDSU), San Diego, California, United States of America; Loyola University Chicago, UNITED STATES

## Abstract

The Caatinga is a semi-arid biome in northeast Brazil. The Paraguaçú River is located in the Caatinga biome, and part of its course is protected by the National Park of Chapada Diamantina (PNCD). In this study we evaluated the effect of PNCD protection on the water quality and microbial community diversity of this river by analyzing water samples obtained from points located inside and outside the PNCD in both wet and dry seasons. Results of water quality analysis showed higher levels of silicate, ammonia, particulate organic carbon, and nitrite in samples from the unprotected area compared with those from protected areas. Pyrosequencing of the 16S rRNA genes revealed that Burkholderiales was abundant in samples from all three sites during both seasons and was represented primarily by the genus *Polynucleobacter* and members of the Comamonadaceae family (e.g., genus *Limnohabitans*). During the dry season, the unprotected area showed a higher abundance of *Flavobacterium* sp. and *Arthrobacter* sp., which are frequently associated with the presence and/or degradation of arsenic and pesticide compounds. In addition, genes that appear to be related to agricultural impacts on the environment, as well as those involved in arsenic and cadmium resistance, copper homeostasis, and propanediol utilization, were detected in the unprotected areas by metagenomic sequencing. Although PNCD protection improves water quality, agricultural activities around the park may affect water quality within the park and may account for the presence of bacteria capable of pesticide degradation and assimilation, evidencing possible anthropogenic impacts on the Caatinga.

## Introduction

The Caatinga is a semi-arid biome located in the northeast of Brazil (3–17°S to 35–45°W). It occupies almost 900,000 km^2^ of the Brazilian territory and is characterized by its vegetation during the dry season, when the leaves fall and white tree trunks and shrubs remain in the landscape [1, 2]. This unique and important biome is strongly affected by anthropogenic processes. It is estimated that about 50% of the Caatinga biome has been modified by activities related to agriculture, livestock, or coal extraction [3–6]. The dry season in the Caatinga is characterized by water shortage [5], with an annual rainfall of 300–500 mm in the semi-arid zone and 1,500 mm in the mountainous area of Chapada Diamantina [3]. Accordingly, the Paraguaçú River, which is a typical Caatinga river, shows high seasonal volume fluctuations. This river supplies water for agricultural and mining activities, as well as several cities. Despite the great importance of this river, it is subject to pesticide dumping, siltation, and heavy metal pollution [7], but the precise effects of agriculture and mining on the water quality and microbial diversity of this region remain unclear. To prevent environmental degradation, the National Park of Chapada Diamantina (PNCD) was created in 1985. However, this park, which protects part of the Paraguaçú River course, is surrounded by agricultural land, some of which is in direct contact with the Paraguaçú River.

Previous studies have described the isolation and characterization of microbes from the Caatinga [8–12]. Recently, Pacchioni *et al*. conducted the first metagenomic study of one soil sample from the Caatinga and showed that Actinobacteria and Alphaproteobacteria were the most abundant groups [13]. Pacchioni *et al*. also described the presence of genes related to stress resistance and the metabolism of DNA, nitrogen, and amino acids. This study suggested that the microbial profile of the Caatinga differed from those of other Brazilian biomes such as the Amazon, savannah (also called Cerrado), and Atlantic forest, but the limited number of samples analyzed hampered a clear distinction. In addition, it is not clear from previous studies how the microbial communities are structured inside and outside protected areas (such as PNCD).

In this study, we evaluated the effect of PNCD protection on the water quality and microbial diversity of the Paraguaçú River. Specifically, we performed taxonomic and metagenomic analyses of bacterial communities from sites inside and outside the PNCD in both wet and dry seasons. We also analyzed water quality by measuring the levels of heavy metals, dissolved organic carbon (DOC), particulate organic carbon (POC), and inorganic nutrients.

## Materials and Methods

### Ethics statement

This study was approved by Instituto Chico Mendes de Conservação da Biodiversidade (ICMBio) in accordance with the Brazilian law (Permit Number: SISBIO31652-1).

### Study area and sample collection

**Fig 1:**
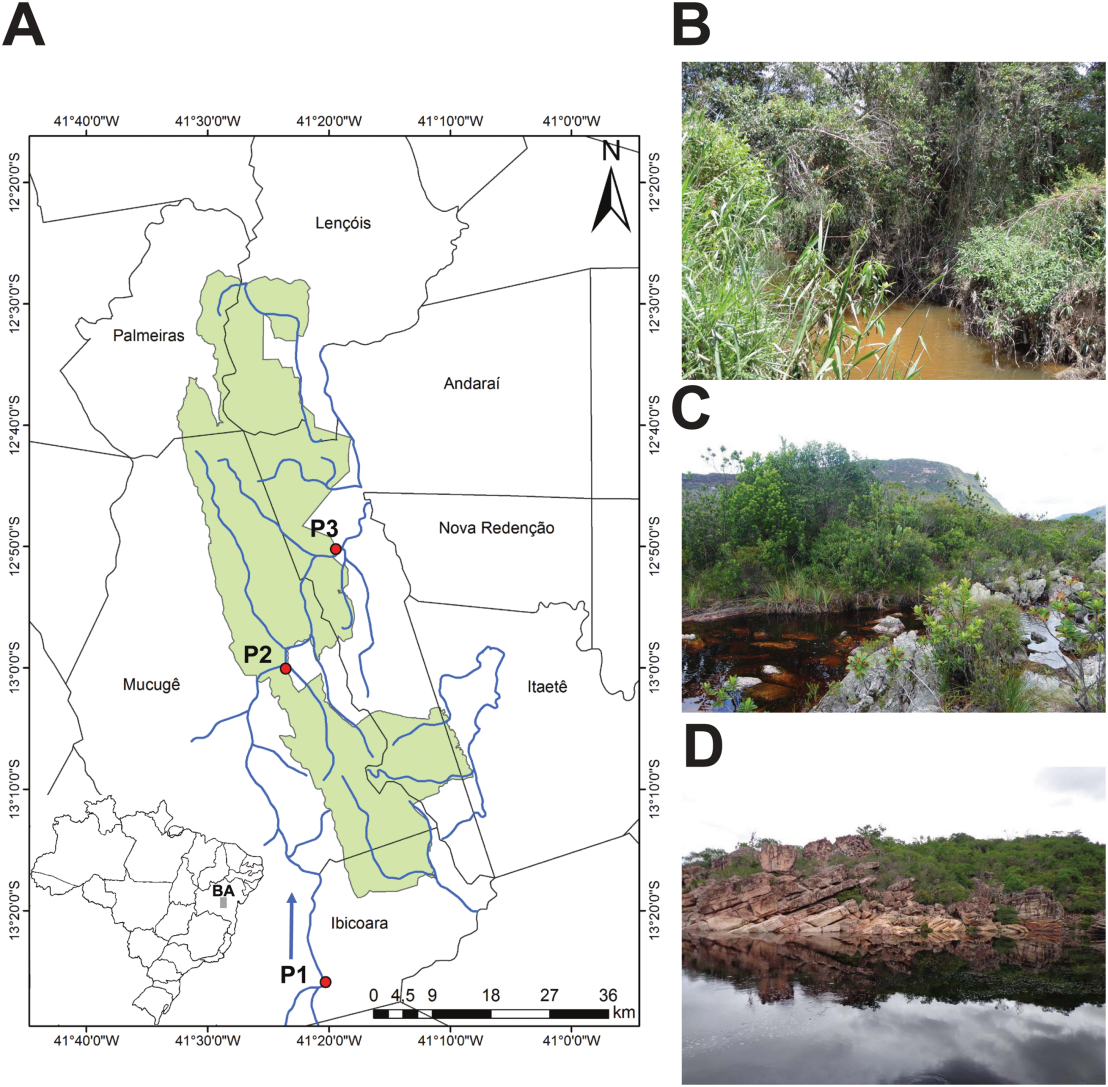
Study area. A) The area under National Park of Chapada Diamantina (PNCD) management is shown in green. The three sampling points on the Paraguaçú River are indicated on the map: P1, unprotected site outside the PNCD; P2 and P3, protected sites within the PNCD. The arrow indicates the direction of water flow. B) Unprotected sampling point P1. C) Protected sampling point P2. D) Protected sampling point P3.

Water samples were collected from three points along the Paraguaçú River (Fig 1). The first sampling point (P1) is located outside the PNCD (13°26'9.11"S41°20'17.56"W) near Ibicoara (Bahia, Brazil) and was therefore unprotected. The two protected sampling points, P2 and P3, are located just inside the PNCD (13°0'2.60"S 41°23'22.57"W and 12°50'25.91"S 41°19'26.52"W, respectively). Two replicate samples were obtained at each sampling point in the wet season (November 2012) and dry season (February 2013) for a total of 12 samples. For each sampling point in both seasons, we collected approximately 20 L unfiltered water by pump from the water column at a depth of approximately 1 m.

After obtaining water samples, aliquots of each sample (200 ml) were frozen at -20°C for the subsequent measurement of physicochemical parameters. After collecting the water samples, sediments were sampled from the river bottom directly below the water sampling point. The sediment samples were collected using a dredge at a depth of 10 cm inside a perimeter of 2 m and stored at -20°C.

For each freshwater sample, aliquots used to measure microbial abundance were prepared immediately after water sampling. Three 1.5-ml aliquots were dispensed into 2.0-ml cryogenic tubes and fixed with 1% paraformaldehyde and 0.5% glutaraldehyde for the bacterial count [14], 0.5% glutaraldehyde for the viral count [15], and 1% paraformaldehyde for the nanoeukaryotic phytoplankton (NEUK), picoeukaryote (PEUK), and *Synechococcus* spp. analyses [16]. Fixation was performed within 30 minutes after the water samples were collected.

Two liters of water was prefiltered using a 20-μm mesh and then filtered through 0.22-μm Sterivex filters (Millipore), using a peristaltic pump and three Sterivex filters for each sample. The Sterivex filters were stored in SET buffer (20% sucrose, 50 mM EDTA, 0.5 mM Tris-HCl, and pH 8.0) at -80°C until DNA extraction could be carried out.

### Physicochemical and microbial abundance analyses

Two replicates water samples were analyzed for each physicochemical parameter. Measurements of inorganic nutrients [17] were carried out as follows: ammonia was determined using the indophenol method, nitrite by diazotization, nitrate by Cd-Cu reduction followed by diazotization, total nitrogen by potassium persulfate digestion following nitrate determination, orthophosphate by reaction with ascorbic acid, total phosphorous by acid digestion to phosphate, and silicate by reaction with molybdate. DOC and POC were analyzed as described previously [18].

Each sediment sample was passed through a 2-mm sieve (Millipore) with a stream of water. Sand (coarse, medium and fine), silt, clay, and trace elements were measured in the fraction that passed through the sieve. Quantification of trace elements was carried out by inductively coupled plasma optical emission spectrometry (ICP-OES; Varian Liberty-Series II) using a procedure based on US Environmental Protection Agency Method 3052 [19] and modified by Marques et al. [20]. Approximately 0.5 g of the sieved sediment was used to determine concentrations of the following elements: Al, Ba, Ca, Cd, Cr, Cu, Fe, Mn, Ni, P, Pb, S, Sr, Ti, V, and Zn. Measurements were carried out in triplicate for each sample, and a coefficient of variation between replicates <10% was considered satisfactory.

Microbial and viral abundance in water samples was determined by flow cytometry (FACSCalibur, BD Biosciences) using nucleic acid affinity fluorophore probes for bacteria [21, 22] and SYBR Green (Life Technologies) for viruses [15]. Picoplankton were detected by the fluorescence of natural photosynthetic pigments [16].

Principal component analysis of physicochemical and microbial parameters was performed using a correlation matrix in Past v.3.01 [23]. Values for physicochemical and microbial parameters from the 12 water samples (two samples taken from three different sites in both seasons) were compared by ANOVA (α < 0.05), followed by the Tukey post hoc test using R statistical software [24].

### Metagenomic DNA extraction

DNA extraction was performed using lysozyme (1 mg/ml) for 1 h at 37°C, as previously described [25]. Then proteinase K (0.2 mg/ml) and 1% sodium dodecyl sulfate (SDS) were added, and the samples were incubated at 55°C for 60 min with gentle agitation. The lysate was rinsed into a new tube with 1 ml SET buffer. Metagenomic DNA was extracted with one volume of phenol:chloroform:isoamyl alcohol (25:24:1) and then precipitated with ethanol and sodium acetate (0.3 M final concentration) at -20°C overnight. The DNA was purified using a Power Clean DNA Clean-Up Kit (MO BIO Laboratories) and stored at -20°C.

### Polymerase chain reaction, 16S rRNA gene amplicon sequencing, and sequence analysis

Polymerase chain reaction (PCR) amplification of bacterial 16S rRNA genes was performed as described in our previous reports [26]. The hypervariable regions V5 to V9 of the bacterial 16S rRNA gene were amplified using primers 787F (5'-ATTAGATACCCNGGTAG-3') and 1492R (5'-GNTACCTTGTTACGACTT-3') [27]. Primers used in this work were designed with the appropriate 454 pyrosequencing adaptor sequences and multiplex identifiers (not shown). Ten 20-μL reactions were carried out for bacteria using 1× buffer, 0.25 mM dNTP mix, 3 mM MgCl_2_, 0.175 pmol each primer (forward and reverse), 1.5 U Taq polymerase (Phoneutria, Brazil), 5–10 ng DNA, and deionized ultrapure water. The PCR conditions consisted of an initial denaturing step at 95°C for 3 min; 25 amplification cycles at 95°C for 30 s, 58°C for 30 s, and 72°C for 1.7 min; and a final extension step for 7 min at 72°C. The reactions were pooled and then purified using a QIAquick PCR Purification kit (Qiagen). Pyrosequencing of the amplicons was carried out in two 1/8 picotiter plates by Macrogen Inc. (Seoul, Korea) using a 454 GS-FLX Titanium system (454 Life Sciences; Roche, Basel, Switzerland).

Sequences were analyzed using QIIME 1.7.0 software [28]. Initially the 16S rRNA sequences were demultiplexed, and the reads were renamed according to the sample ID with the *split_libraries*.*py* script. The sequence quality thresholds used in this step were minimum average quality score of 30, sequence length range of 200–1000 nucleotides, window score of 50 nucleotides, maximum number of ambiguous bases of 6, length of homopolymer run of 6, and maximum number of error in barcodes of 1.5. Primer mismatches were not allowed. The denoising procedure for the output file was performed using the *denoise_wrapper*.*py* script [29] set to titanium defaults. The script *inflate_denoiser_output*.*py* reintegrated the denoised data, and the *truncate_reverse_primer*.*py* script removed the reverse primers and any subsequent sequences. Chimeric sequence identification was performed by *identify_chimeric_seqs*.*py* script using Chimera Slayer [30], which performs reference-based or *de novo* (abundance-based) chimera checking. From a total of 165,337 raw sequences obtained by pyrosequencing, 54,423 sequences were removed after the processing steps described above, yielding 110,914 high-quality sequences.

The *pick_de_novo_otus*.*py* script was used to build an operational taxonomic unit (OTU) table. The parameters used in each step were as follows. (i) OTU picking was performed using uclust with reference sequences from the Greengenes database (May 2013) [31] in the bacterial analyses. Reverse strand matching was enabled, and the sequence similarity threshold was 97%. (ii) The representative set of sequences was chosen based on the most abundant sequences of each OTU. (iii) PyNAST [32] was used to align sequences to the Greengenes core reference alignment, and uclust was used for pairwise alignment; minimum sequence length to include in alignment was 150 nucleotides. (iv) RDP Classifier v.2.2 [33] was used to assign taxonomy based on the Greengenes sequences as references and templates. (v) The phylogenetic tree was built using the FastTree method [34]. (vi) The OTU table showing the relative abundance at each taxonomic level (kingdom, phylum, class, order, family, and genus) was then generated from OTU counts for each sample and their taxonomic assignments.

The script *alpha_rarefaction*.*py* calculated the diversity indices at a single sequencing depth (i.e. number of sequences per sample) with 2910 sequences using the following metrics: nonparametric Shannon [35], Chao1 [36], Observed Species, and Good’s coverage [37]. The values of the indices from the 12 samples (taken from three sites in both seasons with two replicates each) were compared by ANOVA (α < 0.05), followed by the Tukey post hoc test using R statistical software [24]. The script beta_rarefaction.py was used to calculate beta diversity [38]. Results of the principal coordinates analysis (PCoA) of relative abundances of bacterial taxa (at the order and genus levels) were plotted in EMPeror [39] from community similarities values (unweighted UniFrac distance matrices).

We analyzed taxonomic profiles of the water samples using Statistical Analysis of Metagenomic Profiles (STAMP) software [40]. Samples were compared by ANOVA, followed by the Tukey–Kramer post hoc test (p < 0.05) with the Bonferroni correction for multiple comparisons. Taxa with small effect sizes were removed by filtering (effect size = 8.00), and asymptotic confidence intervals (95%) were calculated. For seasonal analysis (wet versus dry), samples were compared by t-test (p < 0.05), followed by the Bonferroni correction for multiple comparisons. Taxa with small effect sizes were removed by filtering (effect size = 8.00), and asymptotic confidence intervals (95%) were calculated.

### Metagenome sequencing and sequence analysis

Metagenomic DNA libraries were constructed with the Nextera DNA Sample Preparation Kit (Illumina) and 2 × 250 bp paired-end sequencing was carried out by Centro de Genômica de Alto Desempenho UCB (Brasília, Brazil) using an Illumina MiSeq system according to the manufacturer's instructions. Sequence analysis was performed with 2.2 x 10^7^ sequences (S1 Table) using the MG-RAST server [41] using default sequence quality thresholds [42, 43]. The analysis of metagenomic data was based on unassembled reads. Functional annotation was performed against the SEED database [44], and taxonomic profiles were generated using the M5NR database [45].

We compared the abundance of gene and taxa among the water samples using STAMP software [40]. Samples were compared by ANOVA, followed by the Tukey–Kramer post hoc test (p < 0.05) without correction for the gene profile and with the Bonferroni correction for multiple comparisons for the taxonomic profile. Genes and taxa with small effect sizes were removed by filtering (effect size = 8.00), and asymptotic confidence intervals (95%) were calculated. For seasonal analysis (wet versus dry; protected versus unprotected in both seasons), samples were compared by t-test (p < 0.05) without correction (gene profile) and with the Bonferroni correction for multiple comparisons (taxonomic profile). Genes and taxa with small effect sizes were removed by filtering (effect size = 8.00).

The phylogenetic analysis was performed from the taxonomy profile provided at the species level by MEGAN software 5.10.6 [46].

### Accession numbers

The sequences assessed in this study are available in NCBI Sequence Read Archive (SRA) under the study Accession number PRJNA292014. Metagenomic data sets are available in the MG-RAST server under Biodiversidade Microbiana do Bioma Caatinga project (ID 7927).

## Results

### Inorganic and organic compounds and microbial cell count

**Fig 2:**
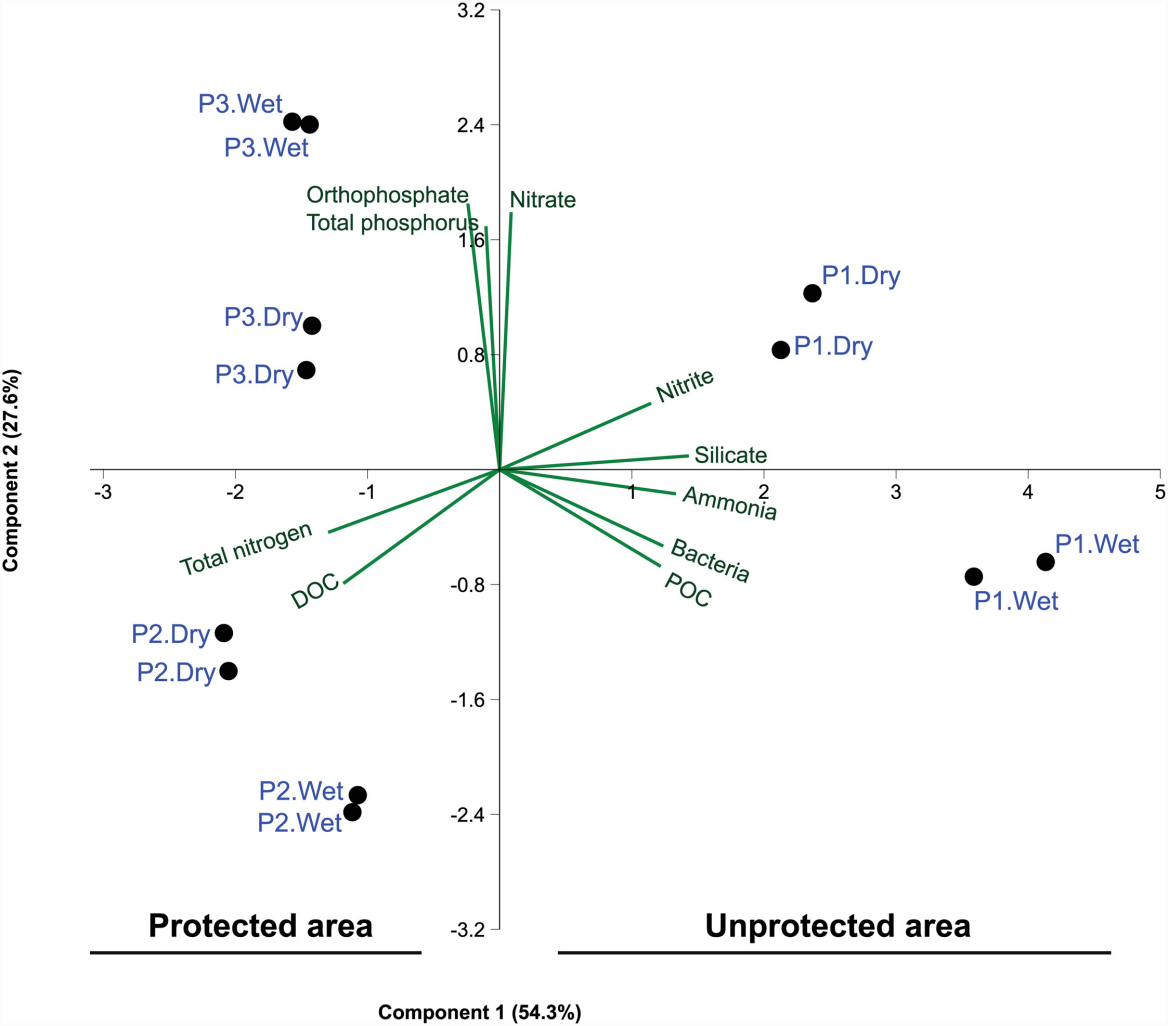
Principal component analysis (PCA) of the physicochemical parameters and bacterial community of Paraguaçú River water samples obtained from three sites (P1, unprotected area; P2 and P3, protected areas) during both wet and dry seasons. The ordination analysis was performed with ammonia, nitrite, nitrate, total nitrogen, orthophosphate, total phosphorous, silicate, dissolved organic carbon (DOC), particulate organic carbon (POC), and bacterial counts based on the correlation matrix.

We tested water samples obtained from an unprotected site outside the PNCD (P1) and two protected areas within the PNCD (P2 and P3) in both wet and dry seasons. Our results showed that levels of silicate, ammonia, POC, and nitrite were higher in samples from the unprotected area, whereas levels of DOC, total nitrogen, orthophosphate, and total phosphorus were higher in samples from the protected areas (Table 1; S1–S3 Figs), allowing the segregation of the samples into two groups (Fig 2). Samples obtained in the unprotected area were also clustered according to season, with the vectors for POC and ammonia grouping samples obtained during the wet season, and vectors for silicate, nitrite, and nitrate grouping samples obtained during the dry season (Fig 2). A comparison of samples obtained from within the PNCD shows that DOC and total nitrogen levels were higher in samples from P2, but total phosphorus and orthophosphate levels were higher in samples from P3 (Fig 2). Table 2 summarizes the water analysis results of our study and those of several other metagenomic studies reporting the physicochemical properties of freshwater samples (e.g. rivers, lakes, reservoirs).

**Table 1:** General features of water samples obtained from sites on the Paraguaçú River.

	P1		P2		P3	
**Geographic location**	13°26'9.11"S	41°20'17.56"W	13° 0'2.60"S	41°23'22.57"W	12°50'25.91"S	41°19'26.52"W
**Altitude (m above sea level)**	1100		974		345	
**Month/Year**	Nov 2012	Feb 2013	Nov 2012	Feb 2013	Nov 2012	Feb 2013
**Season**	wet	dry	wet	dry	wet	dry
**Depth (m)**	± 0.80	± 0.70	± 1.10	± 0.45	> 3.00	> 3.00
**Width (m)**	± 4.23		± 2.94		± 36.9	
**pH**	2.8	4.0	2.9	3.8	3.2	4.1
**Conductivity (μS/ml)**	41	44	54	63	27	35
**Coarse sand (%)**	2.6	5.5	45.7	41.9	68.2	10.5
**Medium sand (%)**	82.3	63.4	48.7	52.4	28.3	78.3
**Fine sand (%)**	14.8	28.9	5.6	5.7	3.3	10.7
**Silt (%)**	0.3	2.2	0.0	0.0	0.0	0.5
**Clay (%)**	0.0	0.0	0.0	0.0	0.0	0.0
**Bacterial counts (cells/ml)**	2.1E+06 ± 1.1E+05	1.2E+06 ± 1.9E+05	1.0E+06 ± 2.0E+04	9.9E+05 ± 6.8E+04	8.7E+05 ± 4.3E+04	7.1E+05 ± 2.4E+04
**Virus counts (CFU/ml)**	6.0E+05 ± 4.3E+04	6.9E+06 ± 1.9E+06	6.6E+05 ± 1.4E+05	1.2E+07 ± 5.6E+05	3.7E+05 ± 1.2E+05	8.7E+06 ± 4.2E+05
***Synechococcus* (cells/ml)**	0 ± 0	0 ± 0	0 ± 0	2.2E+03 ± 5.0E+02	0 ± 0	0 ± 0
**PEUK (cells/ml)**	0 ± 0	0 ± 0	1.6E+03 ± 2.4E+02	3.1E+03 ± 1.7E+02	1.6E+03 ± 6.2E+01	2.0E+03 ± 9.7E+01
**NEUK (cells/ml)**	0 ± 0	0 ± 0	0 ± 0	0 ± 0	0 ± 0	0 ± 0
**DOC (mg/l)**	0.90 ± 0.06	1.25 ± 0.15	2.01 ± 0.14	2.88 ± 0.12	1.52 ± 0.18	1.95 ± 0.09
**POC (mg/l)**	7.53 ± 1.07	2.36 ± 0.17	2.89 ± 0.22	0.71 ± 0.04	0.97 ± 0.11	0.76 ± 0.03
**Orthophosphate (μM)**	0.16 ± 0.01	0.27 ± 0.01	0.05 ± 0.00	0.23 ± 0.01	0.37 ± 0.00	0.24 ± 0.03
**Total phosphorus (μM)**	0.36 ± 0.02	0.28 ± 0.02	0.13 ± 0.01	0.25 ± 0.03	0.70 ± 0.01	0.34 ± 0.01
**Silicate (μM)**	16.03 ± 0.11	11.64 ± 0.98	0.66 ± 0.01	0.29 ± 0.04	1.35 ± 0.01	0.56 ± 0.01
**Ammonia (μM)**	3.20 ± 0.10	2.55 ± 0.11	1.07 ± 0.05	1.53 ± 0.09	0.83 ± 0.06	1.56 ± 0.05
**Nitrite (μM)**	0.31 ± 0.01	0.35 ± 0.01	0.28 ± 0.00	0.27 ± 0.01	0.28 ± 0.01	0.28 ± 0.00
**Nitrate (μM)**	3.25 ± 0.15	5.77 ± 0.20	0.45 ± 0.01	0.69 ± 0.03	6.52 ± 0.11	8.03 ± 0.12
**Total nitrogen (μM)**	28.19 ± 0.76	21.25 ± 0.53	43.82 ± 0.16	46.65 ± 1.28	42.08 ± 1.25	44.15 ± 0.89
**N/P ratio**	79.05 ± 4.81	75.39 ± 4.07	338.88 ± 27.32	189.57 ± 20.05	60.17 ± 1.86	130.27 ± 1.71

Abbreviations: CFU, colony-forming units; DOC, dissolved organic carbon; NEUK, nanoeukaryotes; N/P, nitrogen/phosphorus; PEUK, picoeukaryotes; POC, particulate organic carbon.

**Table 2:** Results of freshwater metagenomics studies.

Study	Freshwater classification	Name	Sample	Observation	Bacterial counts (cells/ml)	Depth (m)	pH	DO (mg/l)	Conductivity (mS/cm)	Salinity (PSU)	DOC (mg/l)	POC (mg/l)	Orthophosphate (μM)	Total phosphorus (μM)	Silicate (μM)	Ammonia (μM)	Nitrite (μM)	Nitrate (μM)	Total nitrogen (μM)	N/P ratio
**Present study**	River	Paraguaçú	**P1**	Wet season	21.49. 10^5^	± 0.80	2.80		0.04		0.9	7.5	0.2	0.4	16	3.2	0.3	3.3	28.2	79.1
			**P2**	Wet season	10. 10^5^	± 1.10	2.90		0.05		2	2.9	0.1	0.1	0.7	1.1	0.3	0.4	43.8	338.9
			**P3**	Wet season	8.73. 10^5^	> 3.00	3.20		0.03		1.5	1	0.4	0.7	1.4	0.8	0.3	6.5	42.1	60.2
			**P1**	Dry season	11.88. 10^5^	± 0.70	4.00	4.64	0.04		1.2	2.4	0.3	0.3	11.6	2.6	0.3	5.8	21.2	75.4
			**P2**	Dry season	9.86. 10^5^	± 0.45	3.80	4.7	0.06		2.9	0.7	0.2	0.2	0.3	1.5	0.3	0.7	46.6	189.6
			**P3**	Dry season	7.13. 10^5^	> 3.00	4.10	6.21	0.04		1.9	0.8	0.2	0.3	0.6	1.6	0.3	8	44.2	130.3
**Liu et al. (2015) [47]**	River	Pearl Estuary	**P01**	Surface water		1	7.01	0.22		0			4.8			139.7	14.8	78.6		
			**P03**	Surface water		1	7.24	3.47		0			2			21.4	24.6	102.5		
			**P07**	Surface water		1	6.99	0.72		0			2			34.4	43.1	105		
			**P01**	Bottom water		7	7.01	0.51		0			4.7			140.1	14.7	96.1		
			**P03**	Bottom water		3	7.21	3.14		0			2.2			30.6	26.4	103		
			**P07**	Bottom water		19	6.98	0.62		0.1			2			31.1	41.9	106.4		
**Yan et al. (2015) [48]**	River	Three Gorges Reservoir	**XXR_E**	Lacustrine system, Xiangxi River estuary			8.24	7.96	374.9				2.1	0.9		0.7		29.4	7.6	
			**XXR_M**	Lacustrine system, Xiangxi River midstream			8.83	12.26	332.3				1.9	1		0.8		15.3	5.3	
			**WJB**	Lacustrine system, Wujia Bay			9.28	19.43	266.8				0.1	0.6		0.2		4.4	3.8	
			**XXR_U**	Riverine system, Xiangxi River upstream			9.52	16.42	282.5				4.1	1.9		0.2		1.5	3.1	
			**BSR_E**	Riverine system, Baisha River estuary			9.25	15.01	266.1				6.8	3.1		0.2		1.3	3.6	
			**SDR_E**	Riverine system, Shendu River estuary			9.24	16.43	271.4				4.6	2.3		0.3		0.4	4.3	
**Tseng et al. (2013) [49]**	Reservoir	Feitsui Reservoir (FTR)	**M1**	Located in North Taiwan	32.46. 10^5^		9.08	7.64	0.09	0.04			0.1				0.3	13.8		
			**M2**	Located in North Taiwan	34.44. 10^5^		8.09	9.45	0.08	0.04			0.1				0.3	17.6		
			**M3**	Located in North Taiwan	23.21. 10^5^		6.52	7.99	0.06	0.03			0.1				0.1	61		
			**M4**	Located in North Taiwan	45.76. 10^5^		8.78	7.35	0.08	0.04			0				0.3	40.2		
			**M5**	Located in North Taiwan	35.33. 10^5^		9.13	6.92	0.08	0.04			0				0.3	35.8		
			**M6**	Located in North Taiwan	13.85. 10^5^		6.96	6.99	0.06	0.04			0				0.1	37.6		
**Ghai et al. (2011) [50]**	River	Solimões-Amazon		Whitewater streams with high sediment concentrations		8	7.09	5.05	109.7											
**Hemme et al. (2010) [51]**	Groundwater	Oak Ridge	**FW301**	Pristine groundwater			± 7.00											6.5		
		Field Research Center	**FW106**	Uranium contamination	10^4^–10^5^	± 12.00	3.70	0.26										10046.6		

Abbreviations: DO, dissolved oxygen; DOC, dissolved organic carbon; N/P, nitrogen/phosphorus; POC, particulate organic carbon; PSU, practical salinity units.

We also found that sand and silt percentages and bacterial and eukaryotic cell counts differed among sampling points (Table 1). Although none of the samples contained clay, P1 had a lower percentage of coarse sand than the other sites and was the only site with silt during both seasons. Picoeukaryotes were detected only in the protected areas (in both seasons), whereas *Synechococcus* was detected only in P2 in the dry season (Table 1). Total virus counts were higher in samples obtained during the dry season than in those obtained during the wet season (S1B Fig). In addition, bacterial cell counts were higher in samples obtained from P1 than in samples from the protected sampling points (Fig 2 and S1A Fig). Analysis of trace elements is shown in S2 Table.

### Microbial community structure

**Fig 3:**
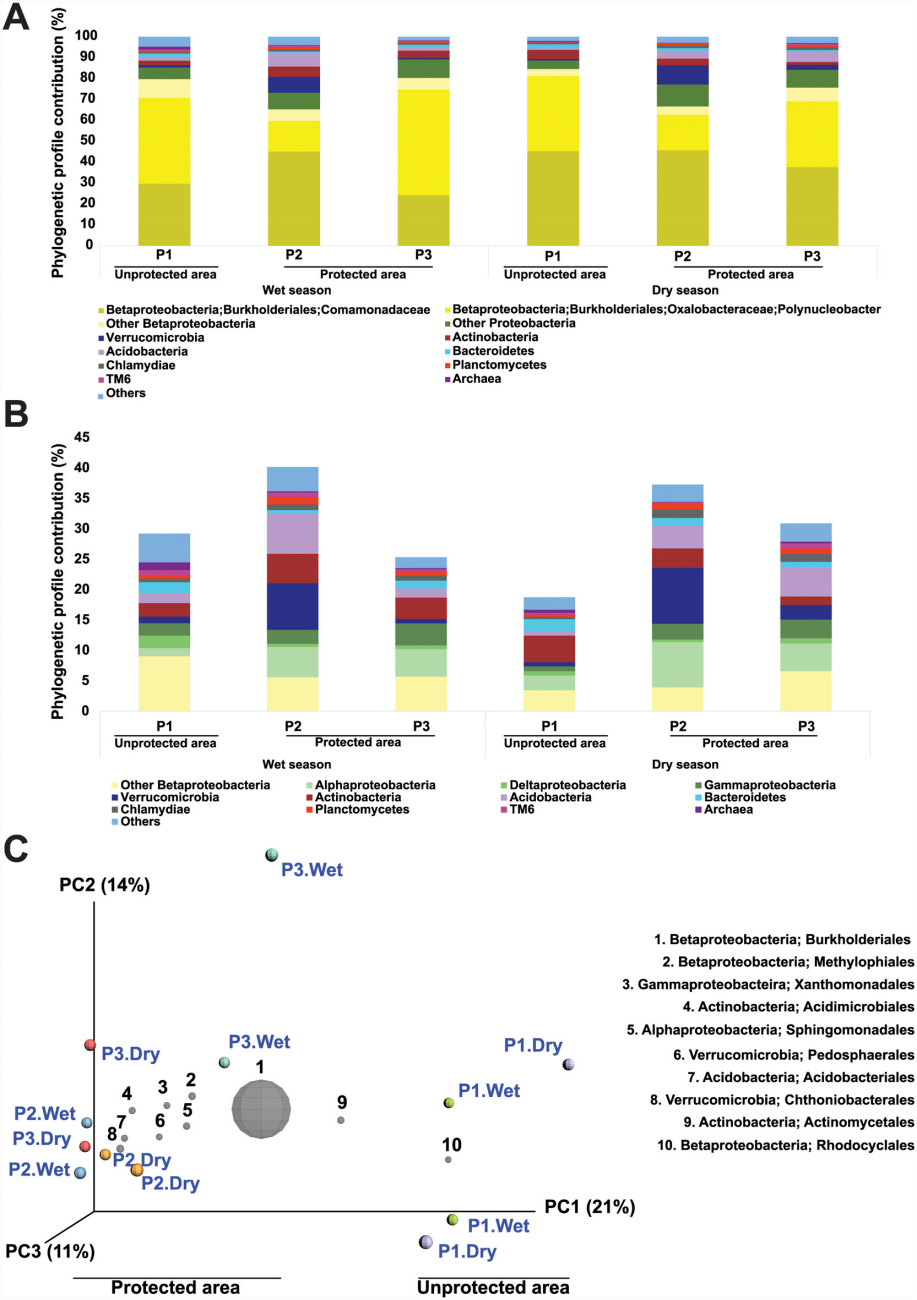
Taxonomic classification based on sequence analysis of 16S rRNA genes detected in Paraguaçú River water samples obtained from three sampling points (P1, unprotected area; P2 and P3, protected area) during both wet and dry seasons. A) Total relative abundance of taxonomic groups (kingdom, phylum, class, family, and genus). B) Relative abundance of taxonomic groups (kingdom, phylum, and class) without the contribution of the Comamonadaceae family and *Polynucleobacter* genus. C) Principal coordinates analysis (PCoA) of relative abundances of taxonomic groups (at the order level). PCoA plot of community similarities (unweighted UniFrac distance matrices). The relative abundance of each taxonomic level (kingdom, phylum, class, and order) for each sample and their taxonomic assignments were determined by QIIME 1.7.0 software with the Greengenes database (May 2013).

The 454 pyrosequencing of the 16S rRNA gene revealed similar profiles at high taxonomic levels (phylum and some classes) among the samples (Fig 3 and S3 Table), and statistical differences were not found between any of the diversity indices tested (S4 Table). The phylum Proteobacteria (primarily Betaproteobacteria) accounted for 79.54% of the OTUs and was observed in all samples analyzed (Fig 3A). Burkholderiales, an order within Betaproteobacteria, was most frequently detected, contributing 70% of all OTUs (S3 Table). The OTUs from this order belonged mainly to the families Comamonadaceae (represented by the genus *Limnohabitans* and other genera) and Burkholderiaceae (represented by the genus *Polynucleobacter*). The abundance of Comamonadaceae was similar across sampling points during the wet season (P1 (30.09%), P2 (44.38%), P3 (24.10%), p > 0.05) and dry season (P1 (45.44%), P2 (46.07%), P3 (36.60%), p > 0.05). The abundance of the genus *Limnohabitans* was similar between seasons (p > 0.05) and across sampling sites (p > 0.05): P1 (0.81% and 2.29% in the wet and dry seasons, respectively), P2 (2.88% and 1.53%), and P3 (1.65% and 1.04%). The abundance of the genus *Polynucleobacter* was also similar between seasons (p > 0.05) and across sampling sites (p > 0.05): P1 (40.96% and 35.82% in the wet and dry seasons, respectively), P2 (14.6% and 16.98%), and P3 (50.43% and 31.42%). In contrast, genus *Flavobacterium* sp. (phylum Bacteroidetes) was more abundant during the dry season in P1 than in P2 and P3 (p < 0.05, S4 Fig). The other orders within Betaproteobacteria, such as Methylophilales and candidate division MND1, comprised 5.71% of the total sequences.

Other classes of Proteobacteria that were detected in our samples included Gammaproteobacteria and Deltaproteobacteria, which represented about 2.4% and 0.86% of the sequences, respectively (Fig 3B and S3 Table). The class Alphaproteobacteria accounted for about 4.2% of total sequences. Verrucomicrobia was the second most abundant phylum, comprising about 3.63% of the total sequences. The phyla Actinobacteria and Acidobacteria were less abundant (3.25% and 3.18% of the total sequences, respectively). Taken together, the relative abundance of the other phyla was about 7.0% (Fig 3B and S3 Table). No taxonomical differences were found between the wet and dry seasons.

The microbial structure of samples obtained from the protected areas differed from that of the unprotected area (Fig 3C). Although the relative abundance of Burkholderiales (Betaproteobacteria) was similar across samples, the three sampling points were clustered according to order. The following orders were more abundant in the protected areas P2 and P3: Methylophilales (Betaproteobacteria), Xanthomonadales (Gammaproteobacteria), Acidimicrobiales (Actinobacteria), Sphingomonadales (Alphaproteobacteria), Pedosphaerales (Verrucomicrobia), Acidobacteriales (Acidobacteria), and Chthoniobacterales (Verrucomicrobia) (Fig 3C). On the other hand, the orders Actinomycetales (Actinobacteria) and Rhodocyclales (Betaproteobacteria) were more abundant in the unprotected area P1. No segregation was observed according to season (dry versus wet). A similar pattern was seen in the OTU-level analysis (S5 Fig). However, the OTU-level analysis shows the influence of genus Candidatus *Rhodoluna* (order Actinomycetales) and genus C39 (order Rhodocyclales) in the clustering of samples from the unprotected area P1.

The taxonomic profile from metagenomic data (S6 Fig) revealed that *Polynucleobacter necessaries* was the species most frequently detected, contributing 17.12% of all OTUs (S5 Table). The most abundant genera according to percentage of total OTUs were *Polynucleobacter* (17.12%), *Acidovorax* (5.94%), *Burkholderia* (3.22%), *Polaromonas*, (3.07%), and *Ralstonia* (1.80%). Differences among samples were observed only in the dry season, during which *Arthrobacter* sp. was more abundant in P1 than in P2 and P3 (S7 Fig).

### Functional classification of metagenome data

**Fig 4:**
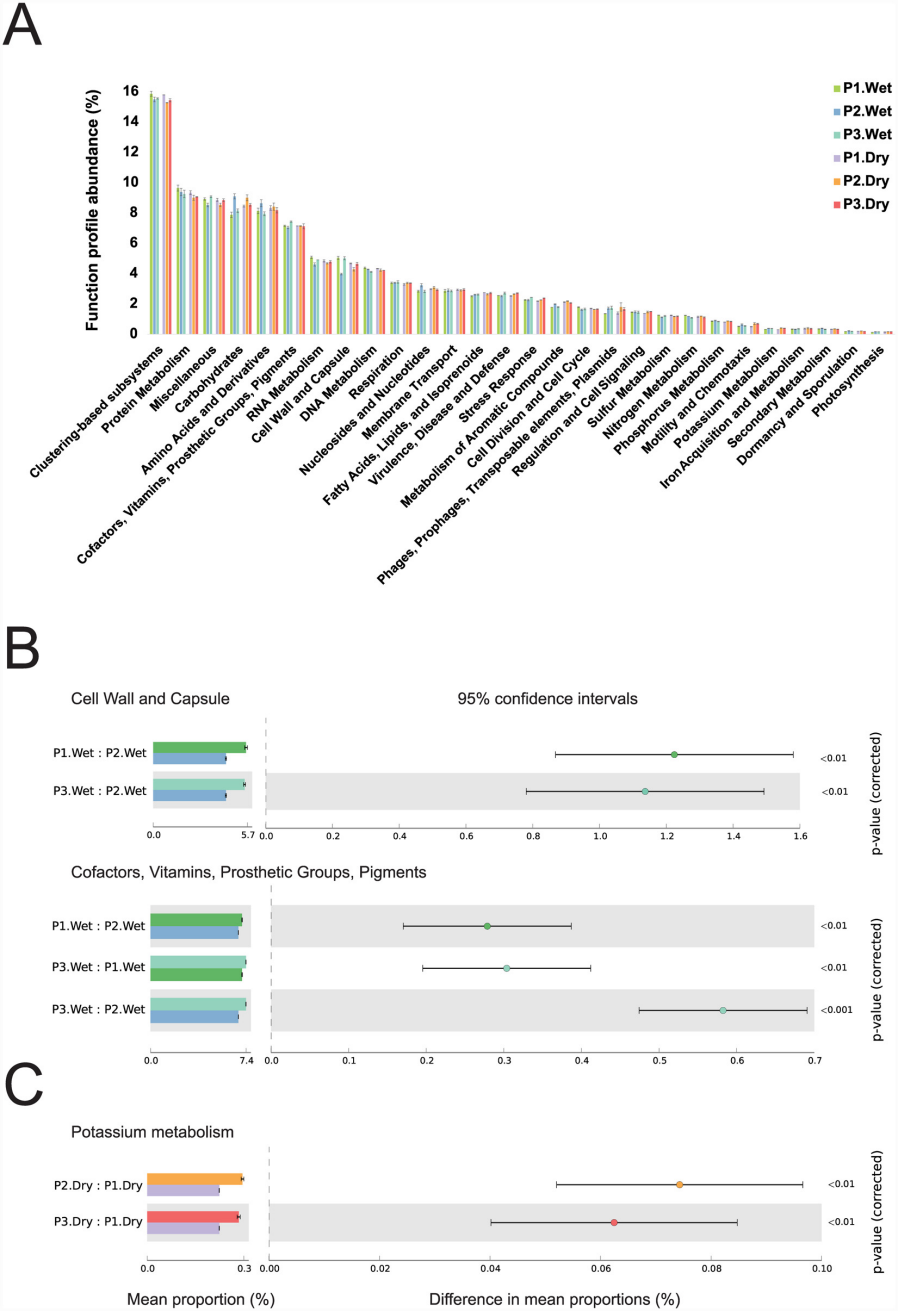
Functional diversity of the Paraguaçú River’s metagenomes (P1, unprotected area; P2 and P3, protected area) in both wet and dry seasons. Classification was based on the SEED database level 1 in the MG-RAST server. A) Relative abundance of genes grouped by functional role. B) Comparative analysis of functional profiles of Paraguaçú River water samples obtained in the wet season. C) Comparative analysis of functional profiles of Paraguaçú River water samples obtained in the dry season. Samples were compared by t-test (p < 0.05), followed by the Bonferroni correction using STAMP software.

After quality control filtering, approximately 82.54% of the sequences (1.56 × 10^7^) were annotated with an assigned function using the SEED database (Fig 4A). Most of the annotated sequences (51.4%) were assigned to one of the following categories: protein metabolism; carbohydrates; amino acids and derivatives; cofactors, vitamins, prosthetic groups, pigments; RNA metabolism; or miscellaneous. Proteins with unknown function accounted for about 15.5% of the annotated sequences. In the wet season, sequences assigned to the cell wall and capsule category and the cofactor, vitamins, prosthetic groups, pigments category were more abundant in sampling points P1 and P3 than in P2 (Fig 4B), whereas in dry season sequences related to potassium metabolism were more abundant in P2 and P3 than in P1 (Fig 4C). A comparison of samples by season showed that annotated sequences related to the metabolism of aromatic compounds were more abundant in the dry season (S8 Fig).

**Fig 5:**
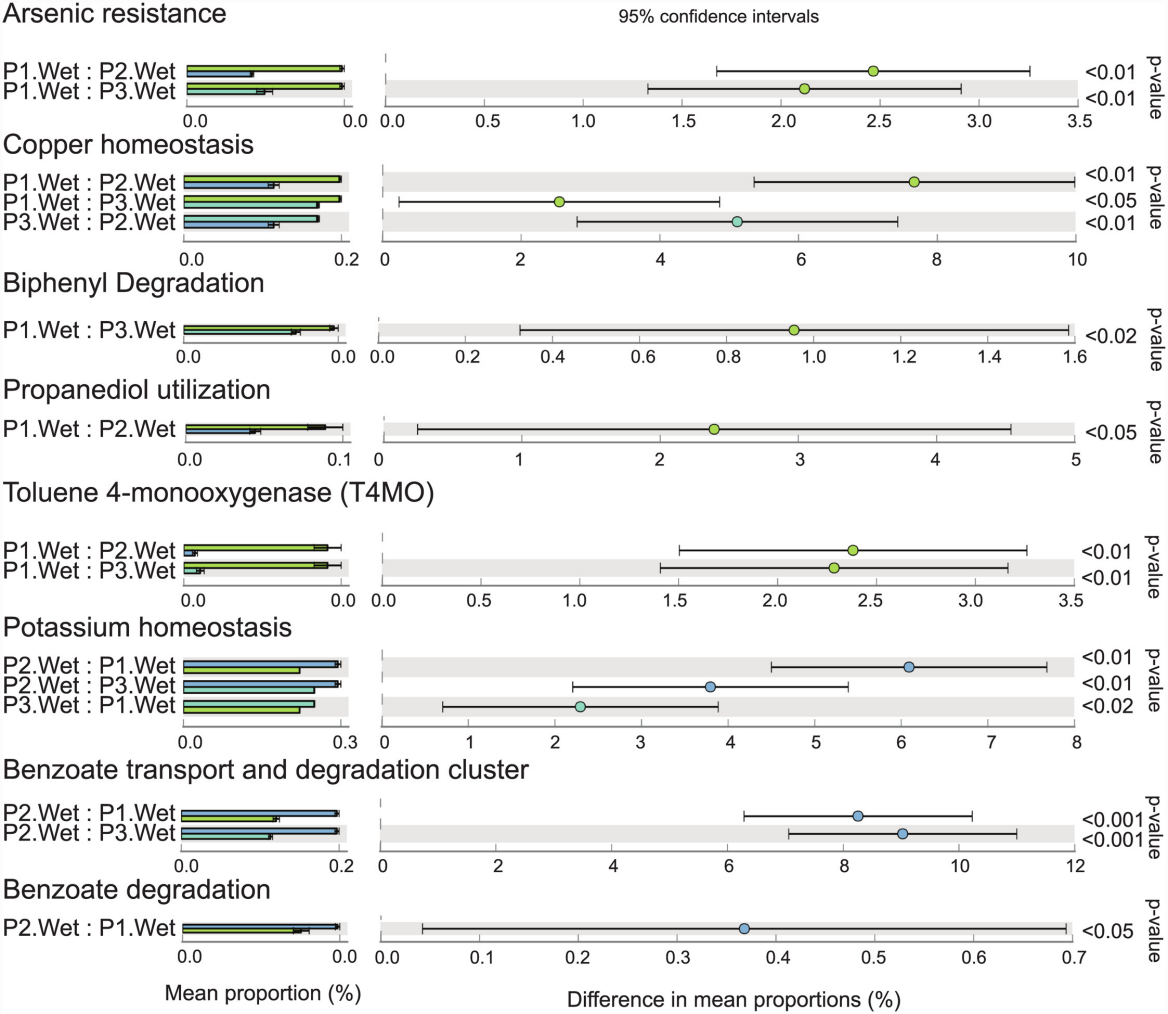
Comparative analysis of pesticide-related genes in Paraguaçú River water samples obtained in the wet season. Samples were compared by ANOVA, followed by the Tukey–Kramer post hoc test (p < 0.05) without correction for multiple comparisons. Taxa with small effect sizes were removed by filtering (effect size = 8.00) using STAMP software.

**Fig 6:**
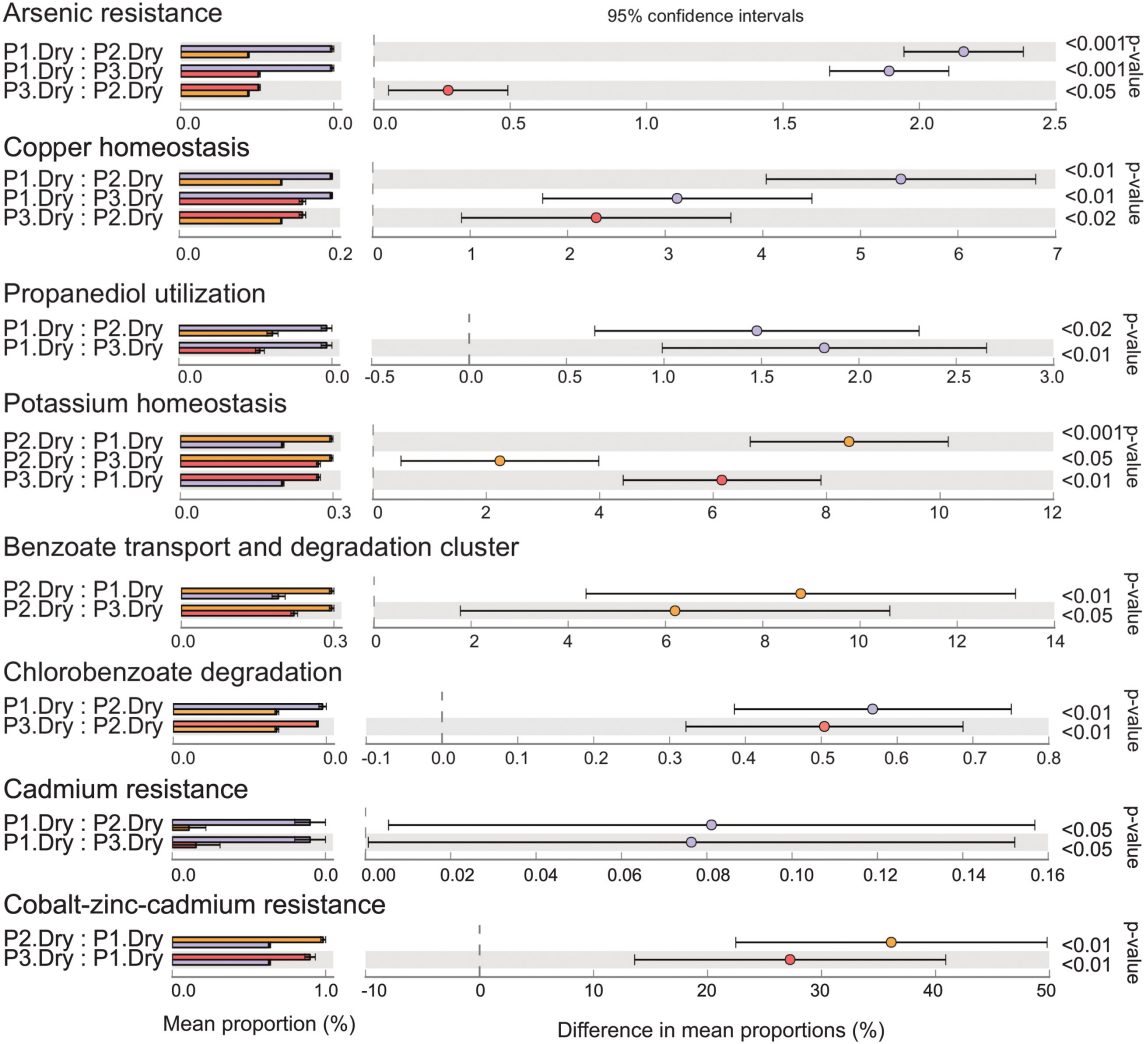
Comparative analysis of pesticide-related genes in Paraguaçú River water samples obtained in the dry season. Samples were compared by ANOVA, followed by the Tukey–Kramer post hoc test (p < 0.05) without correction for multiple comparisons. Taxa with small effect sizes were removed by filtering (effect size = 8.00) using STAMP software.

Although they were not abundant, several genes that appear to be related to pesticide degradation were detected (Figs 5 and 6). In both seasons, genes related to arsenic resistance, copper homeostasis, and propanediol utilization were more abundant in P1. Genes related to biphenyl degradation and toluene 4-monooxygenase (T4MO) were also more abundant in P1 but only during the wet season (Fig 5). Genes related to cadmium resistance were more abundant in P1 during the dry season (Fig 6).

In contrast, the protected area P2 showed a higher abundance of genes related to potassium homeostasis and the benzoate transport and degradation cluster in both seasons, compared with the other sampling sites (Figs 5 and 6). Similarly, genes related to cobalt-zinc-cadmium resistance were more abundant in both protected areas during the dry season compared to the unprotected area. However, the abundance pattern of genes involved in the chlorobenzoate degradation pathway was different, with higher abundance during the dry season in P1 and P3 compared with P2.

Genes related to nitrogen, sulfur, and phosphorus metabolism were also evaluated (S9 and S10 Figs). Regarding genes involved in nitrogen uptake and assimilation (S9A and S10A Figs), ammonia assimilation, nitrogen fixation, and nitrosative stress genes were more abundant in P1 during the wet season. The relative abundance of nitrate and nitrite ammonification genes was higher in P2 than in P3 during the wet season, but was higher in P2 than in P1 during the dry season. During the dry season, urea decomposition and urease subunits genes were most abundant in P2.

Regarding sulfur-related genes (S9B and S10B Figs), P1 showed the highest abundance of inorganic sulfur assimilation and alkanesulfonate utilization in the wet season and the highest abundance of sulfur oxidation and alkanesulfonate utilization genes in the dry season. Although sulfur oxidation genes were more abundant in P1 than in P2 in the dry season, genes for alkanesulfonate assimilation were more abundant in P2 in the wet season.

Finally, regarding phosphorus metabolism (S9C and S10C Figs), genes involved in alkyphosphonate utilization were most abundant in P3 in wet season. In the dry season, genes involved in phosphate metabolism were more abundant in P2 than in P1.

## Discussion

The high abundance of Burkholderiales in samples obtained from all three sampling sites in this study suggests that these microbes are members of the natural microbiome of the Paraguaçú River. However, we cannot rule out that the abundance of this group is due to i) runoff from agriculture lands or ii) enrichment of nutrients and presence of pesticides that could promote their growth in the river. Some Burkholderiales members (e.g. genus *Burkholderia)* are associated with plant roots [52] and could be related to disease resistance or plant disease [53]. *Polynucleobacter* (Burkholderiaceae) and *Comamonas* (Comamonadaceae), which form symbiotic relationships with plants, are found in fresh water [54, 55] and possess genes related to the degradation of pesticides used in agriculture [56]. The genus *Limnohabitans* (family Comamonadaceae) prefers freshwater environments that are nonhumic and nonacidic (pH > 6) [57]. *Limnohabitans* often coexists with genus *Polynucleobacter*, which prefers acidic habitats (pH < 6), but they show different abundances according to the habitat features [58].

Although no differences were observed between ecological parameters of samples collected in the dry season, the genus *Flavobacterium* (phylum Bacteroidetes) was more abundant in samples collected outside the PNCD (P1) in the dry season. The *Flavobacterium* genus includes both nonpathogenic species and several species that cause fish diseases such as columnaris disease, which is caused by *Flavobacterium columnare* and typically occurs in warm waters (20°C –25°C) [59]. This genus has also been detected in arsenic-contaminated groundwater [60].

The protected sampling points located just inside the PNCD (P2 and P3) exhibited lower nutrient concentrations in both wet and dry seasons compared with the unprotected location (P1), which is near agriculture land and may therefore be affected by chemical fertilizers (S11 Fig). The concentrations of several nutrients (e.g. ammonia, nitrite, nitrate, and POC) and the bacterial cell count were highest in samples obtained from P1. Soil fertilization is a common practice in agriculture to increase plant development, but if those nutrients reach the river, as is frequently observed in the wet season, they can stimulate bacteria growth and eutrophication, promoting changes in biodiversity [61, 62]. The process of eutrophication can render unfeasible the use of water for drinking and agricultural activities (e.g. irrigation) because of the presence of bacterial toxins (e.g. microcystin, saxitoxin) [63]. Eutrophication also affects several food webs, thereby affecting nutrient cycling [64]. Several genes related to the utilization of nutrients such as nitrogen (wet season) and sulfur (both seasons) were more abundant in P1 than in the protected sites. Agricultural management could also increase the level of particulate organic matter (POM), quantified by POC, in the river. Microorganisms, which have been shown to colonize POM particles for use as a carbon source, play a role in POM dynamics, leading to increased POM levels in the water [65]. Thus our findings suggest a microbial community response to agriculture. In addition, the high amount of copper in Paraguaçú River sediment at the unprotected (P1) site suggests the presence of agricultural pesticides. Copper can persist in the environment for many years and can be toxic to aquatic organisms in high concentrations [66]. Genes related to copper homeostasis indicate a possible influence on the microbiota (e.g. development of copper resistance) [67]. Furthermore, the genus *Ralstonia* (family Burkholderiaceae) was one of the most abundant genera detected. The species *Ralstonia pickettiia* has been reported in areas with high concentrations of copper, iron, nickel, and zinc and is able to degrade aromatic hydrocarbons and chlorinated phenolic compounds [68–70]. In the protected sites, the scarcity of available organic carbon and other compounds (e.g. ammonia and nitrite) may explain, at least in part, the increase in stress response genes during the dry season (S12 Fig).

Pesticide-related genes, such as those involved in arsenic resistance, propanediol utilization, biphenyl degradation, T4MO, and cadmium resistance, were more abundant in P1 than in the protected sites. These genes are related to the degradation of compounds commonly used in agriculture such as fungicides, bactericides, pesticide solvents, and fertilizers [71–77]. Moreover, our results showed a higher abundance of the genus *Arthrobacter* in P1 in the dry season. *Arthrobacter* (phylum Actinobacteria) is able to degrade atrazine, one of the most commonly used pesticides in several countries. Several studies have described the bioremediation of atrazine-contaminated soil with *Arthrobacter* spp. [78, 79]. Other genera detected include *Acidovorax* and *Polaromonas* (both from family Comamonadaceae), which are known for their arsenite-oxidizing abilities [60, 80]. Genes related to sulfur oxidation and alkanesulfonate utilization were also more abundant in P1 in both seasons, suggesting that agricultural activities increase the amount of sulfonate compounds in the environment and lead to their accumulation in freshwater [81]. Several of these sulfur-related genes were identified as belonging to Actinomycetales, and the presence of Candidatus *Rhodoluna* (order Actinomycetales) helps separate the unprotected sampling site (P1) from the protected sites (P2 and P3). The most protected site (P3) showed lower levels of pesticide-related genes than the other sampling points, indicating the efficacy of PNCD protection. However, even with PNCD protection, signs of anthropogenic activity were observed within the park. For example, P2 had a relatively high abundance of benzoate-related and potassium-related genes. Benzoate is an anion present in emamectin benzoate, an insecticide used against the Lepidoptera *Helicoverpa armigera* [82]. This compound can persist in the environment by binding to particulate material or surfaces, with detrimental effects in aquatic environments [83, 84]. Potassium is used in agriculture because of its importance in several physiological processes in plants and in the water-holding capacity of soils [85]. Runoff may be a source of agriculture-related benzoate and potassium, influencing the bacterial community present in this theoretically protected environment.

We compared our results with those of other metagenomic studies reporting the physicochemical properties of freshwater samples. Despite evidence of anthropogenic actions in the PNCD, water quality parameters of the Paraguaçú River are more similar to those of the Feitsui reservoir [49] than the Pearl estuary [47]. The Feitsui reservoir supplies water to several cities and is used to generate electricity, reflecting its relatively good water quality, whereas the Pearl estuary is intensely polluted with sewage and industrial waste. However, few parameters were evaluated across all studies, making detailed comparisons difficult.

This is the first metagenomic study conducted within the PNCD, which is part of the Caatinga biome. Our results showed important changes in microbial community structure and gene content in an unprotected area near the park, illustrating the importance of park protection to maintain microbial diversity and water quality. Although the PNCD provides some protection of water quality, agricultural activities around the park are still able to affect water quality within the park and may account for the presence of bacteria capable of pesticide degradation and assimilation. Thus, the present study provides evidence of the anthropogenic impact on the Caatinga and demonstrates the need for additional protection of areas near the border of PNCD.

## Supporting Information

S1 Fig Microorganism counts and organic carbon content in water samples obtained from the Paraguaçú River. A) Bacterial counts. B) Virus counts. C) Particulate organic carbon (POC). D) Dissolved organic carbon (DOC). Samples were compared by ANOVA (α < 0.05), followed by the Tukey post hoc test using R statistical software.
(TIF)

S2 Fig Nitrogen content in water samples obtained from the Paraguaçú River. A) Ammonia. B) Nitrate. C) Nitrite. D) Total nitrogen. Samples were compared by ANOVA (α < 0.05), followed by the Tukey post hoc test using R statistical software.
(TIF)

S3 Fig Phosphorus and silicate content in water samples obtained from the Paraguaçú River. A) Orthophosphate. B) Total phosphorus. C) Silicate. Samples were compared by ANOVA (α < 0.05), followed by the Tukey post hoc test using R statistical software.
(TIF)

S4 Fig Comparative analysis of 16S rRNA taxonomic profiles of Paraguaçú River water samples obtained in the dry season. Samples were compared by ANOVA, followed by the Tukey–Kramer post hoc test (p < 0.05) and Bonferroni correction for multiple comparisons. Taxa with small effect sizes were removed by filtering (effect size = 8.00) using STAMP software. OTUs were classified at the order level (3% dissimilarity) using the Greengenes database (May 2013) and QIIME 1.7.0 software.
(TIF)

S5 Fig Principal coordinates analysis (PCoA) of relative abundances of taxonomic groups (at the genus level). The relative abundance of each genus for each sample and their taxonomic assignments was performed using QIIME 1.7.0 software and the Greengenes database (May 2013).
(TIF)

S6 Fig Microbial community profile from metagenome sequencing. OTUs were classified at the species level in MG-RAST using the M5NR database with default sequence quality thresholds and assigned with MEGAN 5.10.6.
(PDF)

S7 Fig Comparative analysis of metagenome taxonomic profiles of Paraguaçú River water samples obtained in the dry season. Samples were compared by ANOVA, followed by the Tukey–Kramer post hoc test (p < 0.05) and Bonferroni correction for multiple comparisons. Taxa with small effect sizes were removed by filtering (effect size = 8.00) using STAMP software. OTUs were classified at the order level in MG-RAST using the M5NR database and default sequence quality thresholds.
(TIF)

S8 Fig Functional diversity of the Paraguaçú River’s metagenomes in both wet and dry seasons. Classification was based on the SEED database level 1 in the MG-RAST server. A) Relative abundance of genes grouped by functional role. B) Comparison of genes involved in the metabolism of aromatic compounds in water samples obtained in the wet and dry seasons. Samples were compared by t-test (p < 0.05), followed by the Bonferroni correction using STAMP software.
(TIF)

S9 Fig Comparative analysis of functional profiles of Paraguaçú River water samples obtained in the wet season. Samples were compared by ANOVA, followed by the Tukey–Kramer post hoc test (p < 0.05) without correction for multiple comparisons. Taxa with small effect sizes were removed by filtering (effect size = 8.00) using STAMP software. A) Nitrogen metabolism-related genes. B) Sulfur metabolism-related genes. C) Phosphorus metabolism-related genes.
(TIF)

S10 Fig Comparative analysis of functional profile of Paraguaçú River samples obtained in the dry the season. Samples were compared by ANOVA, followed by the Tukey–Kramer post hoc test (p < 0.05) without correction for multiple comparisons. Taxa with small effect sizes were removed by filtering (effect size = 8.00) using STAMP software. A) Nitrogen metabolism-related genes. B) Sulfur metabolism-related genes. C) Phosphorus metabolism-related genes.
(TIF)

S11 Fig Satellite image of the study area. (TIF)

S12 Fig Functional diversity of the Paraguaçú River’s metagenomes in samples obtained from an unprotected area (P1) and protected areas (P2 and P3) in both wet and dry seasons. Classification was based on the SEED database level 1 in the MG-RAST server. A) Relative abundance of genes grouped by functional role. B) Comparison of genes involved in photosynthesis detected in water samples from the unprotected and protected areas obtained in the wet season. C) Comparison of genes involved in motility and chemotaxis; potassium metabolism; stress response, and virulence, disease, and defense detected in water samples from the unprotected and protected areas obtained in the dry season. Samples were compared by t-test (p < 0.05), followed by the Bonferroni correction using STAMP software.
(TIF)

S1 Table Number of sequences obtained by metagenome sequencing. Metagenomic DNA libraries were constructed with the Nextera DNA Sample Preparation Kit (Illumina) and 2 × 250 bp paired-end sequencing by Illumina MiSeq system according to the manufacturer's instructions.
(DOC)

S2 Table Metal, sulfur, and phosphorus concentrations of sediments in water samples from the Paraguaçú River. ND = Not detected. * Data from one replicate.
(DOC)

S3 Table Relative abundance of OTUs and sequence information of Paraguaçú River microbiota. OTUs were classified at the level of genus using the Greengenes database (May 2013) at 3% dissimilarity performed by QIIME 1.7.0 software.
(XLSX)

S4 Table Diversity indices of water samples obtained from the Paraguaçú River at 3% dissimilarity for the 16S rRNA gene. (DOC)

S5 Table Relative abundance of OTUs and sequence information of Paraguaçú River metagenome. OTUs were classified at the species level using the M5NR database with default sequence quality thresholds performed using the MG-RAST server.
(XLSX)
