# Supplementary figures and images for: Microbial Community Profile and Water Quality in a Protected Area of the Caatinga Biome
Source: PLoS One. 2016 Feb 16;11(2):e0148296. doi: 10.1371/journal.pone.0148296 (PMC4755664; doi:10.1371/journal.pone.0148296)

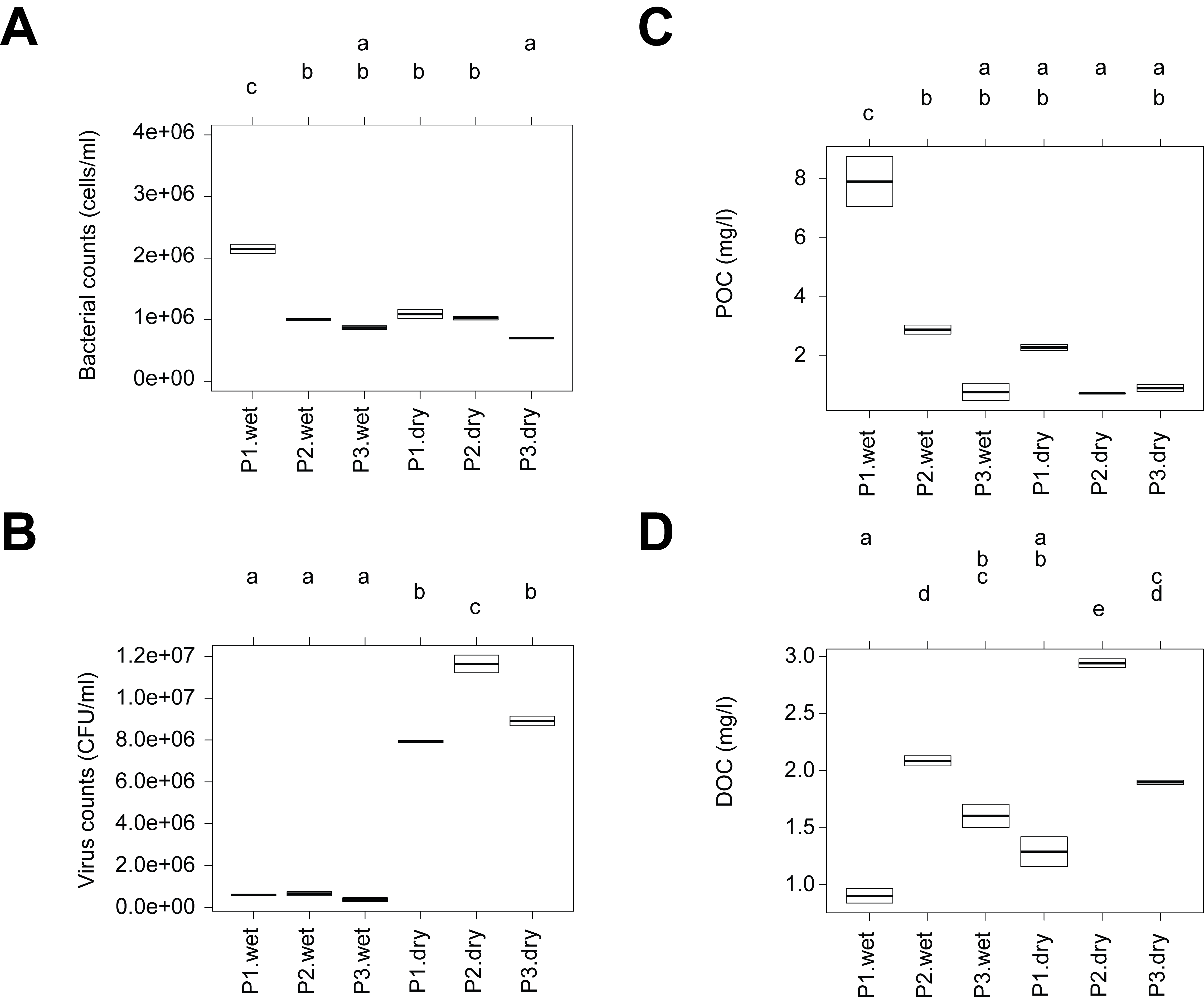

Supplement: S1 Fig — A) Bacterial counts. B) Virus counts. C) Particulate organic carbon (POC). D) Dissolved organic carbon (DOC). Samples were compared by ANOVA (α < 0.05), followed by the Tukey post hoc test using R statistical software. (TIF) [file pone.0148296.s001.tif]

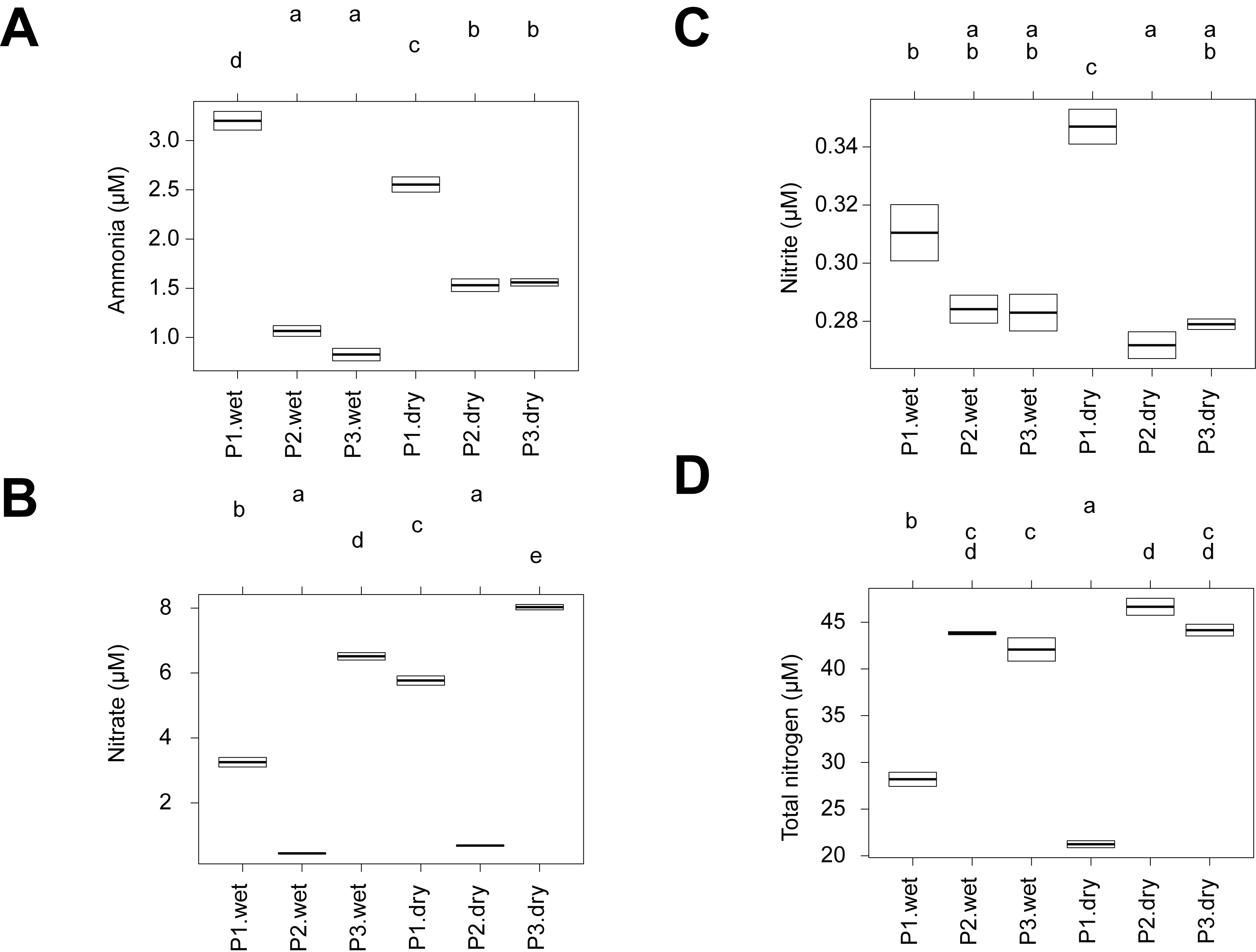

Supplement: S2 Fig — A) Ammonia. B) Nitrate. C) Nitrite. D) Total nitrogen. Samples were compared by ANOVA (α < 0.05), followed by the Tukey post hoc test using R statistical software. (TIF) [file pone.0148296.s002.tif]

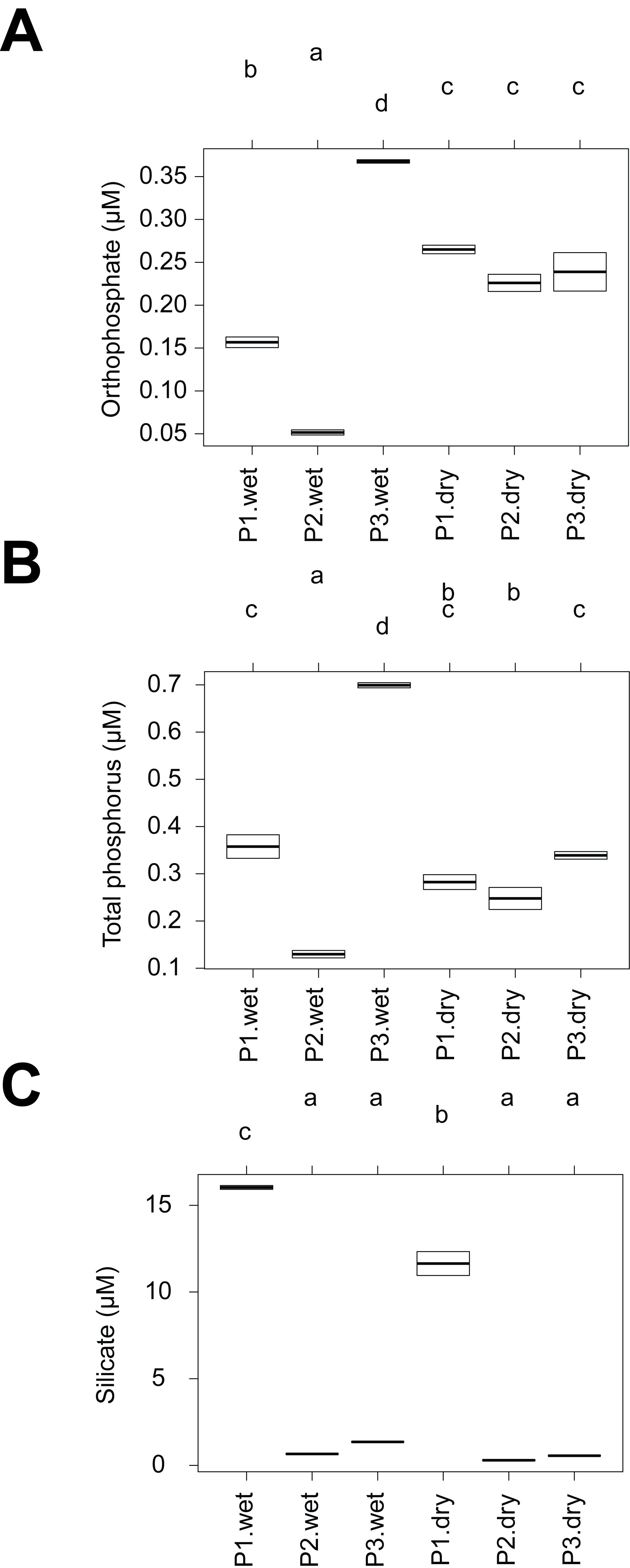

Supplement: S3 Fig — A) Orthophosphate. B) Total phosphorus. C) Silicate. Samples were compared by ANOVA (α < 0.05), followed by the Tukey post hoc test using R statistical software. (TIF) [file pone.0148296.s003.tif]

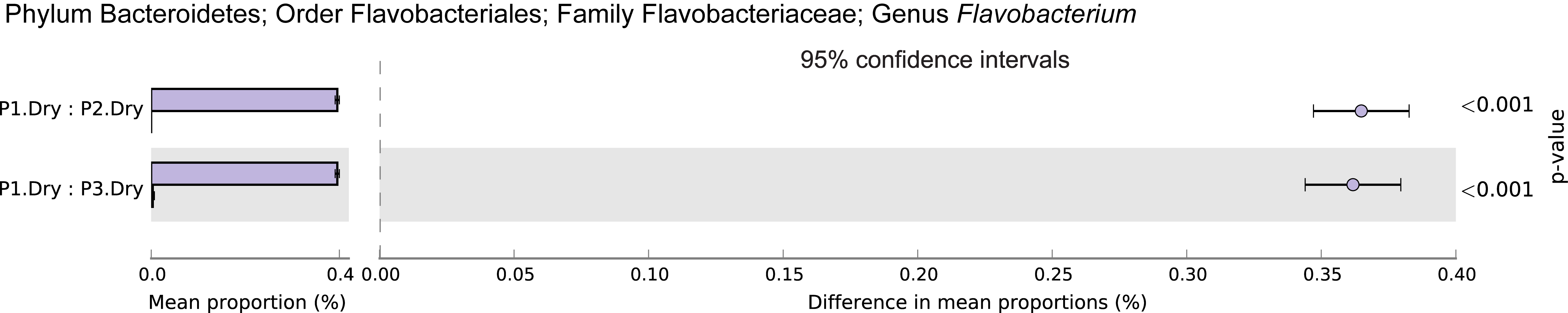

Supplement: S4 Fig — Samples were compared by ANOVA, followed by the Tukey–Kramer post hoc test (p < 0.05) and Bonferroni correction for multiple comparisons. Taxa with small effect sizes were removed by filtering (effect size = 8.00) using STAMP software. OTUs were classified at the order level (3% dissimilarity) using the Greengenes database (May 2013) and QIIME 1.7.0 software. (TIF) [file pone.0148296.s004.tif]

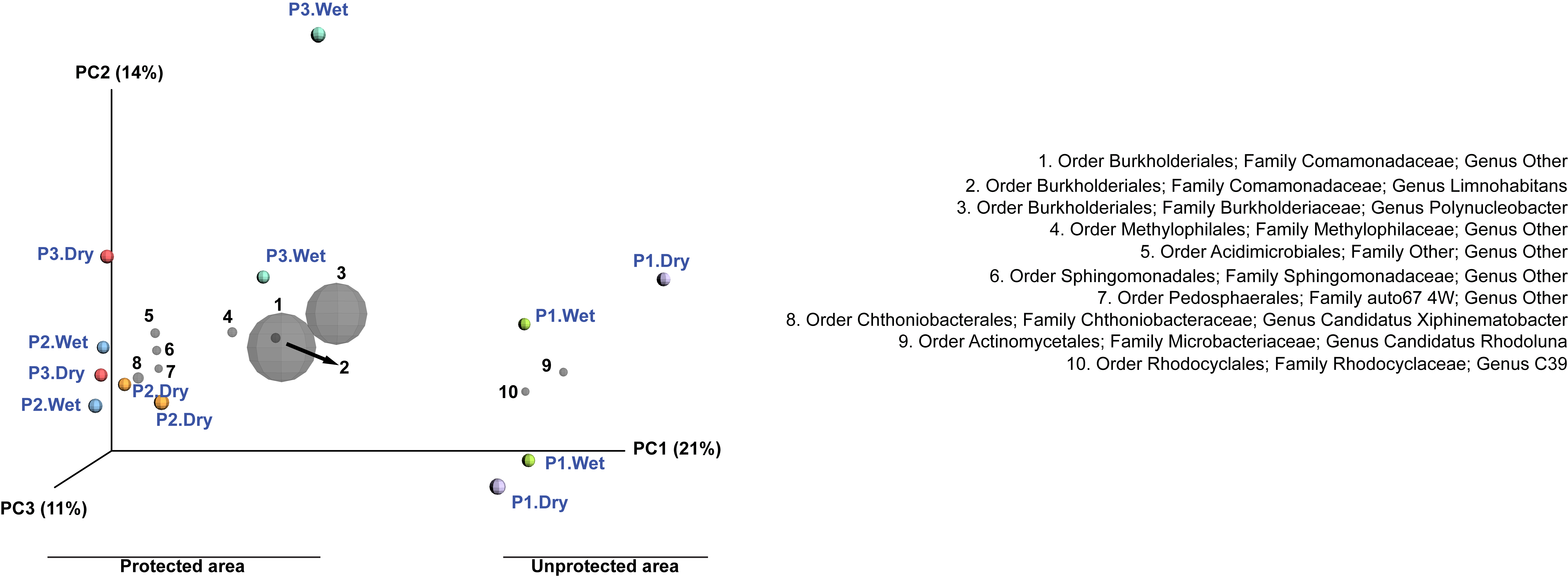

Supplement: S5 Fig — The relative abundance of each genus for each sample and their taxonomic assignments was performed using QIIME 1.7.0 software and the Greengenes database (May 2013). (TIF) [file pone.0148296.s005.tif]

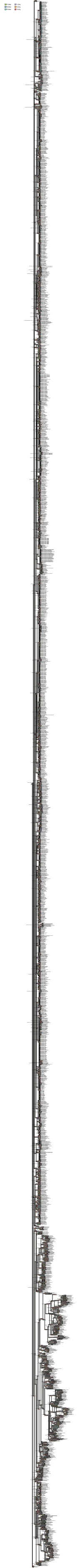

Supplement: S6 Fig — OTUs were classified at the species level in MG-RAST using the M5NR database with default sequence quality thresholds and assigned with MEGAN 5.10.6. (PDF) [file pone.0148296.s006.pdf]

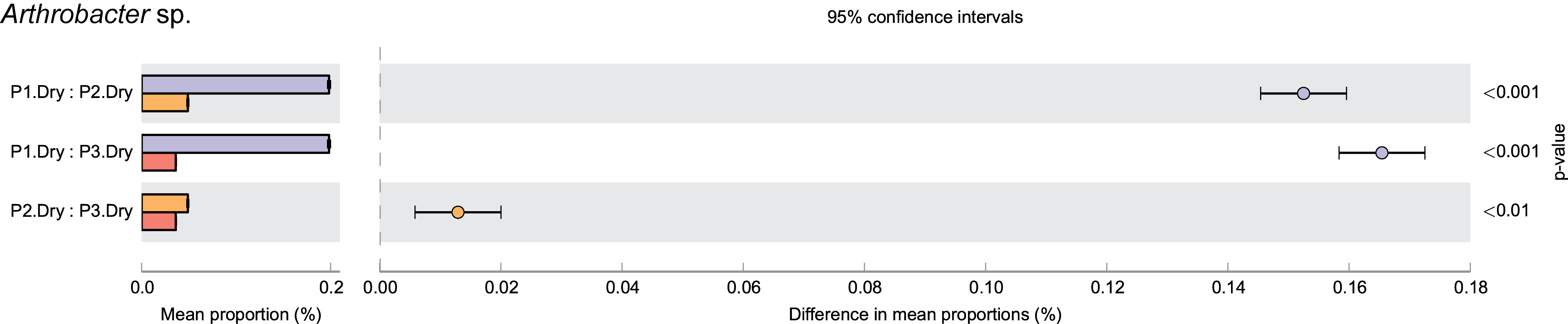

Supplement: S7 Fig — Samples were compared by ANOVA, followed by the Tukey–Kramer post hoc test (p < 0.05) and Bonferroni correction for multiple comparisons. Taxa with small effect sizes were removed by filtering (effect size = 8.00) using STAMP software. OTUs were classified at the order level in MG-RAST using the M5NR database and default sequence quality thresholds. (TIF) [file pone.0148296.s007.tif]

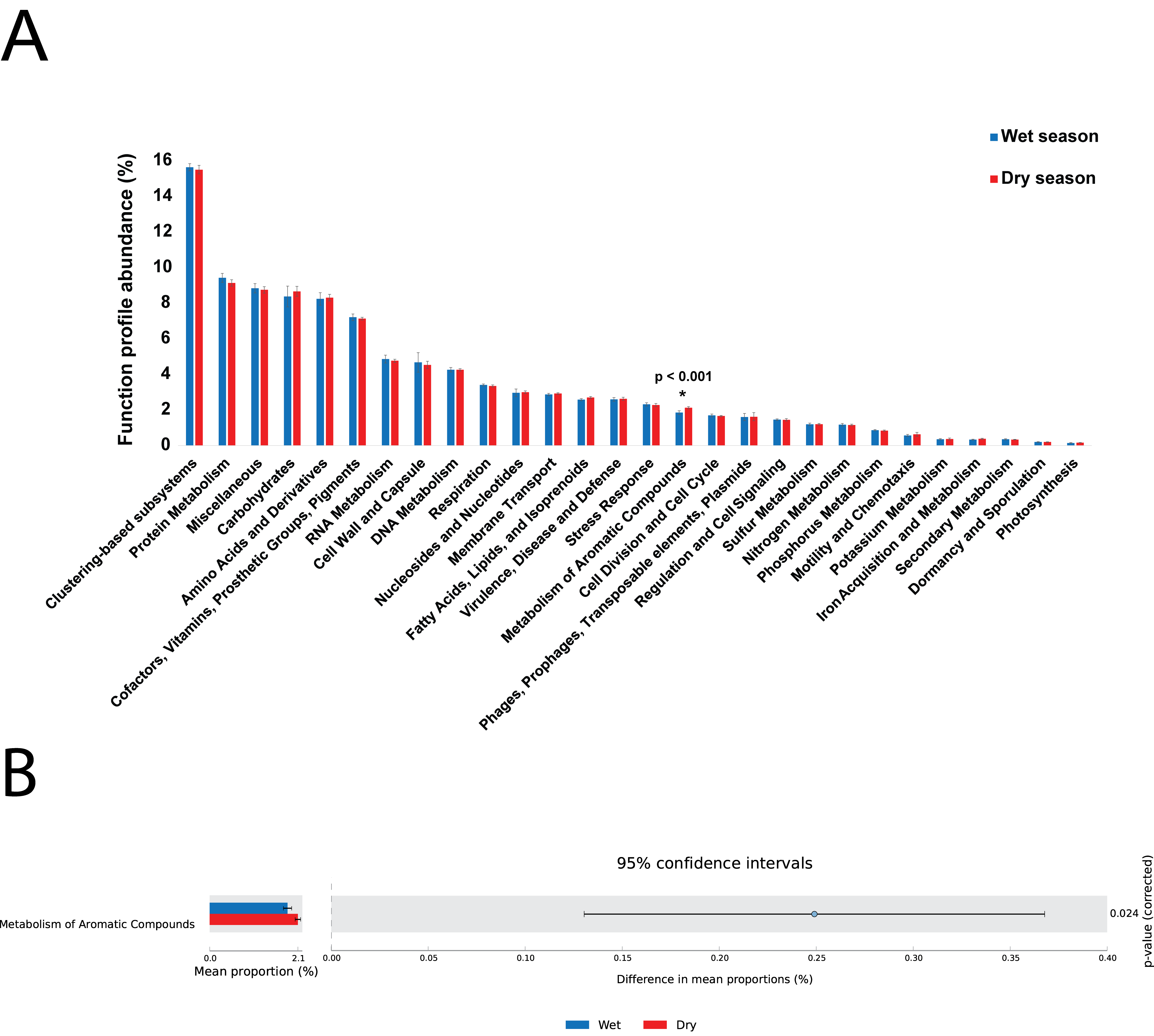

Supplement: S8 Fig — Classification was based on the SEED database level 1 in the MG-RAST server. A) Relative abundance of genes grouped by functional role. B) Comparison of genes involved in the metabolism of aromatic compounds in water samples obtained in the wet and dry seasons. Samples were compared by t-test (p < 0.05), followed by the Bonferroni correction using STAMP software. (TIF) [file pone.0148296.s008.tif]

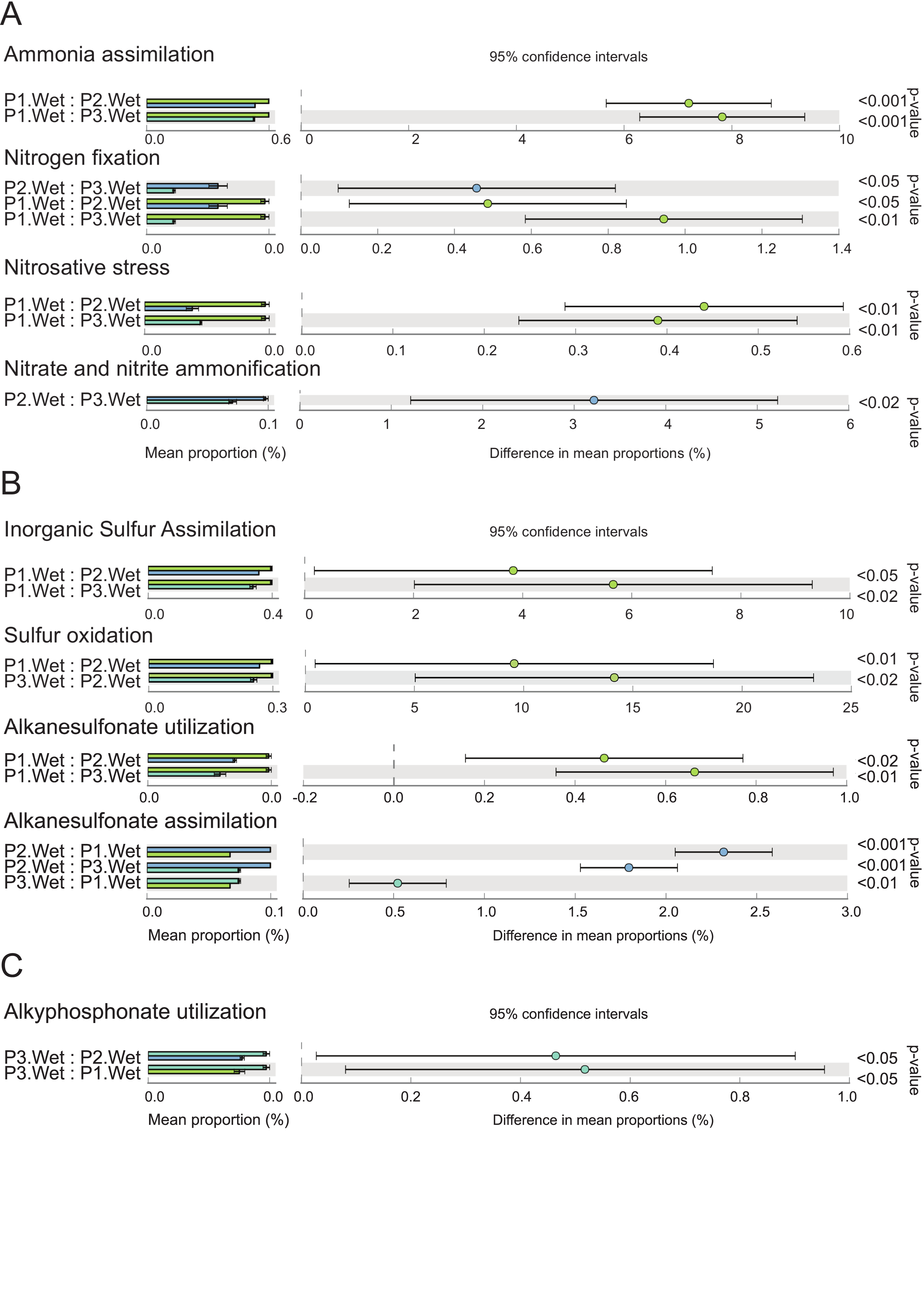

Supplement: S9 Fig — Samples were compared by ANOVA, followed by the Tukey–Kramer post hoc test (p < 0.05) without correction for multiple comparisons. Taxa with small effect sizes were removed by filtering (effect size = 8.00) using STAMP software. A) Nitrogen metabolism-related genes. B) Sulfur metabolism-related genes. C) Phosphorus metabolism-related genes. (TIF) [file pone.0148296.s009.tif]

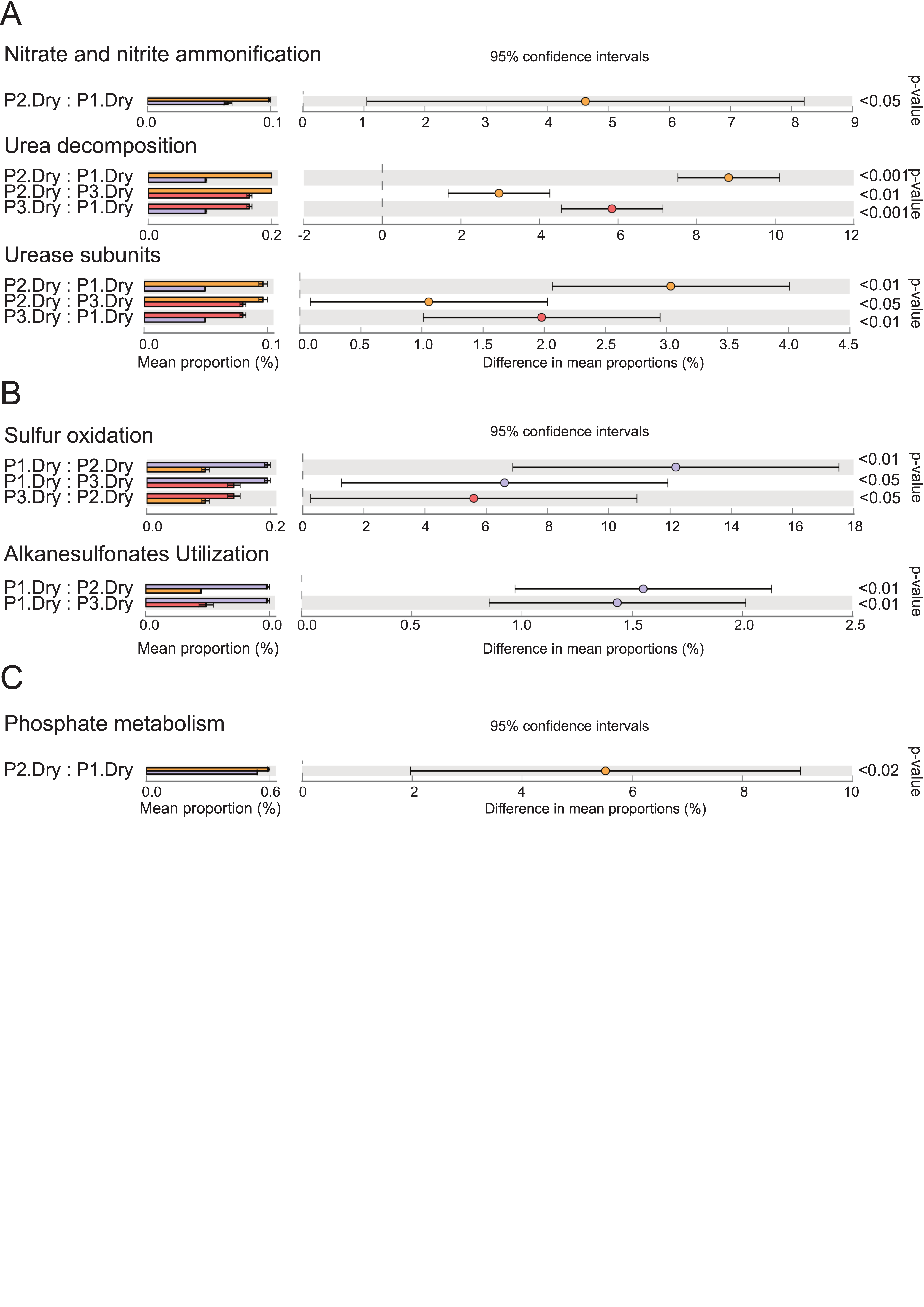

Supplement: S10 Fig — Samples were compared by ANOVA, followed by the Tukey–Kramer post hoc test (p < 0.05) without correction for multiple comparisons. Taxa with small effect sizes were removed by filtering (effect size = 8.00) using STAMP software. A) Nitrogen metabolism-related genes. B) Sulfur metabolism-related genes. C) Phosphorus metabolism-related genes. (TIF) [file pone.0148296.s010.tif]

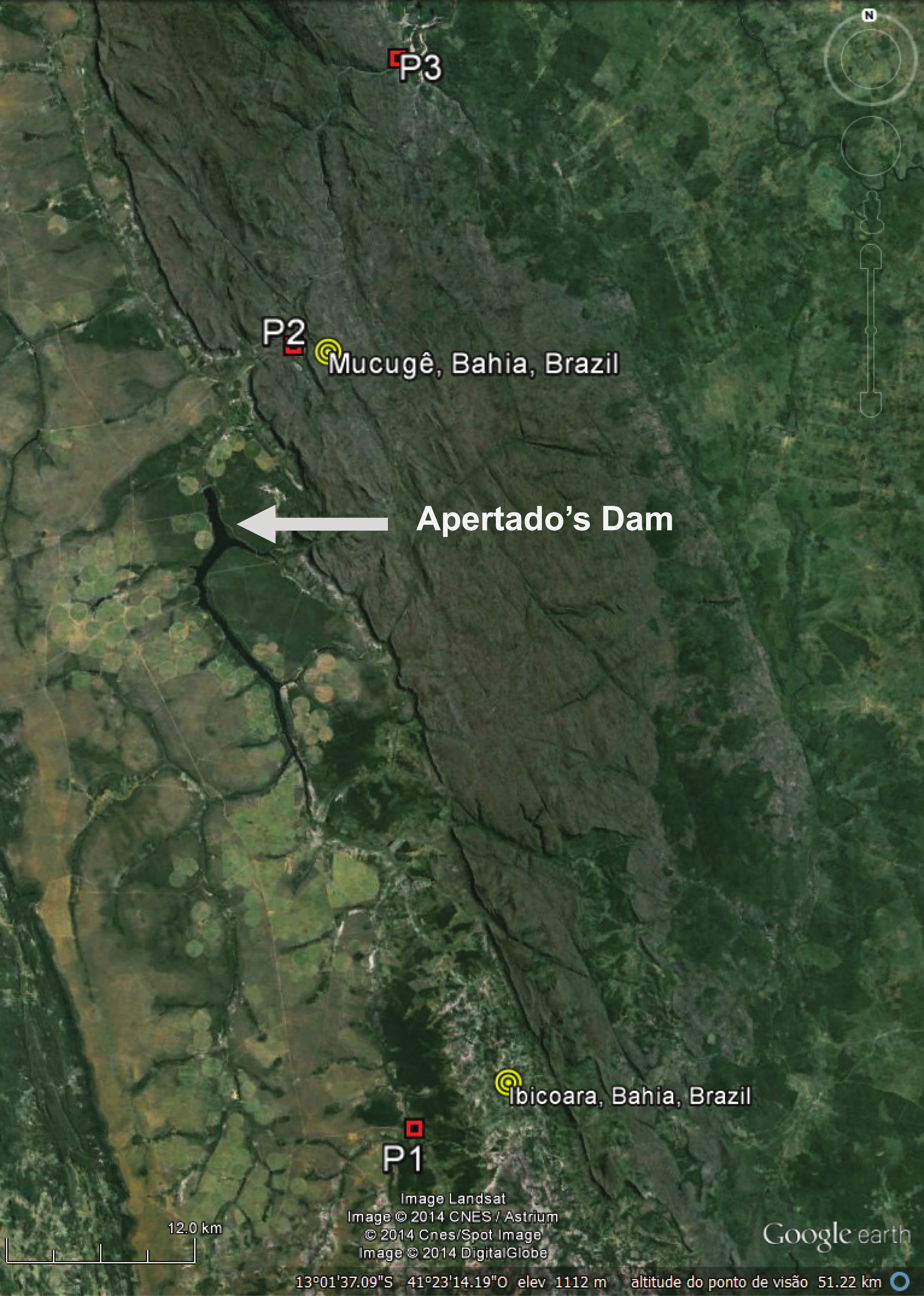

Supplement: S11 Fig — (TIF) [file pone.0148296.s011.tif]

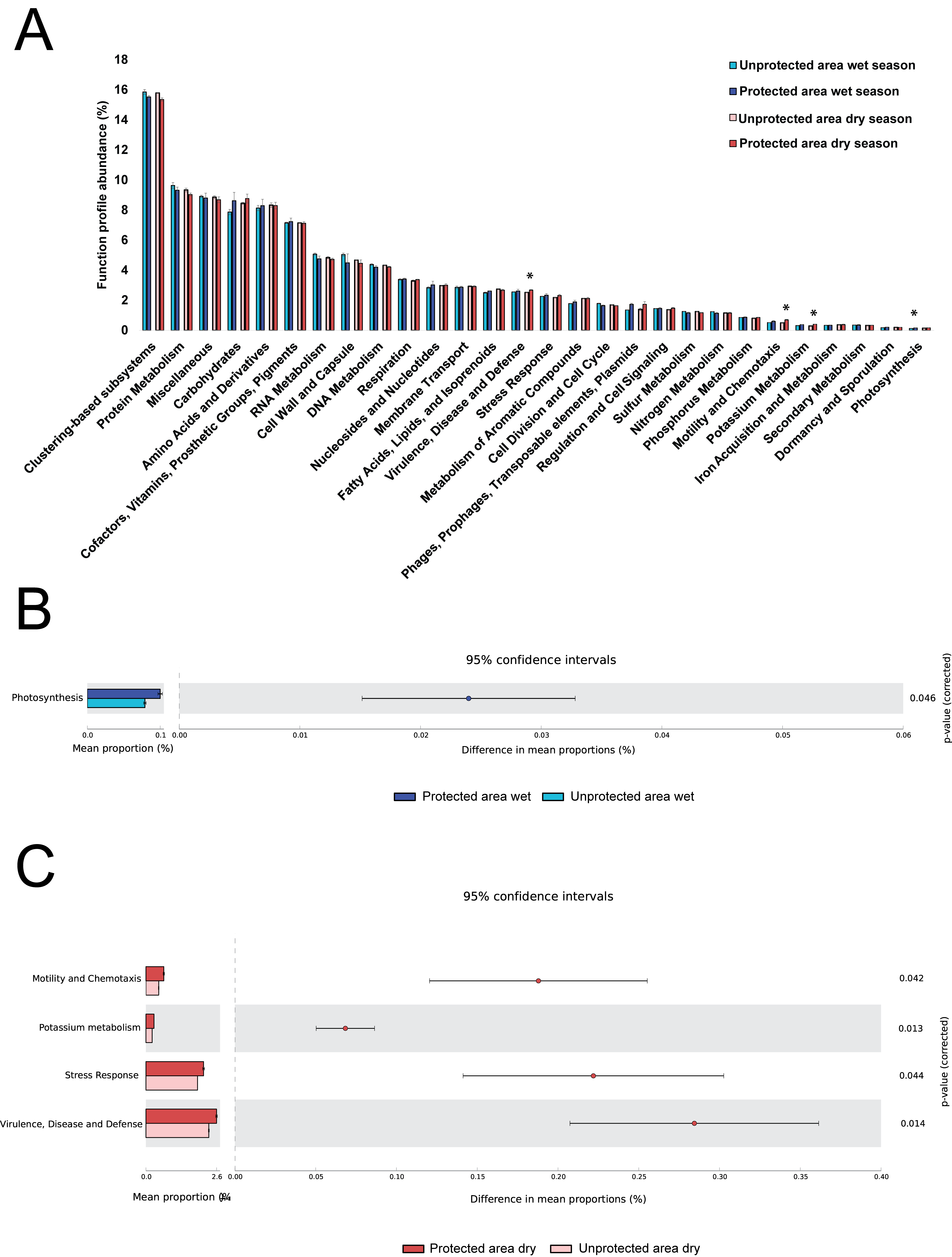

Supplement: S12 Fig — Classification was based on the SEED database level 1 in the MG-RAST server. A) Relative abundance of genes grouped by functional role. B) Comparison of genes involved in photosynthesis detected in water samples from the unprotected and protected areas obtained in the wet season. C) Comparison of genes involved in motility and chemotaxis; potassium metabolism; stress response, and virulence, disease, and defense detected in water samples from the unprotected and protected areas obtained in the dry season. Samples were compared by t-test (p < 0.05), followed by the Bonferroni correction using STAMP software. (TIF) [file pone.0148296.s012.tif]
