# Supplementary material for: Microbial Community Profile and Water Quality in a Protected Area of the Caatinga Biome
Source: PLoS One. 2016 Feb 16;11(2):e0148296. doi: 10.1371/journal.pone.0148296 (PMC4755664; doi:10.1371/journal.pone.0148296)
Supplement: S1 Table — Metagenomic DNA libraries were constructed with the Nextera DNA Sample Preparation Kit (Illumina) and 2 × 250 bp paired-end sequencing by Illumina MiSeq system according to the manufacturer's instructions. (DOC) [file pone.0148296.s013.doc]

**S1 Table. Number of sequences obtained by metagenome sequencing.** Metagenomic DNA libraries were constructed with the Nextera DNA Sample Preparation Kit (Illumina) and 2 × 250 bp paired-end sequencing by Illumina MiSeq system according to the manufacturer's instructions.

| **Season** | **P1.1** | **P1.2** | **P2.1** | **P2.2** | **P3.1** | **P3.2** | **Total** |
| --- | --- | --- | --- | --- | --- | --- | --- |
|  | **(Number of sequences)** | **(Number of sequences)** | **(Number of sequences)** | **(Number of sequences)** | **(Number of sequences)** | **(Number of sequences)** | **(Number of sequences)** |
| **Wet** | 2.60E+06 | 1.00E+06 | 3.00E+06 | 1.10E+06 | 1.90E+06 | 6.20E+05 | 1.00E+07 |
| **Dry** | 3.40E+06 | 1.40E+06 | 2.70E+06 | 1.00E+06 | 2.40E+06 | 7.50E+05 | 1.20E+07 |
| **Total** | - | - | - | - | - | - | 2.20E+07 |
