# Supplementary material for: Microbial Community Profile and Water Quality in a Protected Area of the Caatinga Biome
Source: PLoS One. 2016 Feb 16;11(2):e0148296. doi: 10.1371/journal.pone.0148296 (PMC4755664; doi:10.1371/journal.pone.0148296)
Supplement: S2 Table — ND = Not detected. * Data from one replicate. (DOC) [file pone.0148296.s014.doc]

**S2 Table. Metal, sulfur, and phosphorus concentrations of sediments in water samples from the Paraguaçú River.** ND = Not detected. * Data from one replicate.

| **Compounds** | **P1** | | | | | | **P2** | | | | | | **P3** | | | | | |
| --- | --- | --- | --- | --- | --- | --- | --- | --- | --- | --- | --- | --- | --- | --- | --- | --- | --- | --- |
|  | Nov 2012 | | | Feb 2013 | | | Nov 2012 | | | Feb 2013 | | | Nov 2012 | | | Feb 2013* | | |
| **Al (μg/g)** | 3457.96 | ± | 33.41 | 6121.50 | ± | 1851.28 | 2051.65 | ± | 25.90 | 1143.23 | ± | 61.71 | 3199.14 | ± | 191.82 |  | 3228.818 |  |
| **Ba (μg/g)** |  | ND |  | 10.98 | ± | 1.09 |  | ND |  |  | ND |  |  | ND |  |  | 5.507 |  |
| **Ca (μg/g)** | 51.46 | ± | 5.17 | 41.12 | ± | 8.42 | 29.46 | ± | 0.76 | 23.94 | ± | 4.90 | 58.47 | ± | 2.78 |  | 28.521 |  |
| **Cd (μg/g)** | 0.20 | ± | 0.03 |  | ND |  | 0.24 | ± | 0.01 |  | ND |  | 0.33 | ± | 0.05 |  | ND |  |
| **Cr (μg/g)** | 1.10 | ± | 0.11 | 3.82 | ± | 0.03 | 0.44 | ± | 0.03 | 0.62 | ± | 0.00 | 1.18 | ± | 0.19 |  | 2.248 |  |
| **Cu (μg/g)** | 0.53 | ± | 0.04 | 1.76 | ± | 0.23 |  | ND |  | 0.64 | ± | 0.08 |  | ND |  |  | 0.864 |  |
| **Fe (μg/g)** | 1082.18 | ± | 25.86 | 1666.64 | ± | 443.40 | 778.06 | ± | 120.02 | 88.09 | ± | 86.72 | 668.61 | ± | 63.24 |  | 722.313 |  |
| **Mn (μg/g)** |  | ND |  | 5.63 | ± | 0.07 |  | ND |  | 1.61 | ± | 0.30 |  | ND |  |  | 3.693 |  |
| **Ni (μg/g)** | 1.50 | ± | 0.18 | 2.49 | ± | 0.09 | 1.22 | ± | 0.22 | 2.13 | ± | 0.14 | 1.62 | ± | 0.09 |  | 1.966 |  |
| **P (μg/g)** | 21.06 | ± | 2.50 | 16.97 | ± | 0.91 | 19.73 | ± | 1.75 | 10.86 | ± | 0.02 | 25.96 | ± | 2.93 |  | 23.264 |  |
| **Pb (μg/g)** |  | ND |  | 8.40 | ± | 0.28 |  | ND |  | 8.05 | ± | 0.08 |  | ND |  |  | 7.731 |  |
| **S (μg/g)** | 8.37 | ± | 1.02 | 28.28 | ± | 7.07 | 21.34 | ± | 0.40 | 38.70 | ± | 4.52 | 44.18 | ± | 6.71 |  | 34.001 |  |
| **Sr (μg/g)** |  | ND |  | 2.02 | ± | 0.21 |  | ND |  | 9.33 | ± | 0.61 | 0.54 | ± | 0.32 |  | 17.169 |  |
| **Ti (μg/g)** | 163.41 | ± | 14.46 | 164.32 | ± | 29.57 | 129.51 | ± | 11.43 | 31.77 | ± | 9.17 | 266.63 | ± | 27.33 |  | 310.633 |  |
| **V (μg/g)** | 3.61 | ± | 0.19 | 4.91 | ± | 0.44 | 2.52 | ± | 0.21 | 1.23 | ± | 0.11 | 3.65 | ± | 0.32 |  | 4.490 |  |
| **Zn (μg/g)** | 1.81 | ± | 0.06 | 0.41 | ± | 0.99 | 0.31 | ± | 0.20 | 1.23 | ± | 0.35 | 2.77 | ± | 0.19 |  | 1.564 |  |
