# Supplementary material for: Microbial Community Profile and Water Quality in a Protected Area of the Caatinga Biome
Source: PLoS One. 2016 Feb 16;11(2):e0148296. doi: 10.1371/journal.pone.0148296 (PMC4755664; doi:10.1371/journal.pone.0148296)
Supplement: S4 Table — (DOC) [file pone.0148296.s016.doc]

**S4 Table. Diversity indices of water samples obtained from the Paraguaçú River at 3% dissimilarity for the 16S rRNA gene.**

| **Samples** | **Season** | **OTUs observed** | | | **Chao1** | | | **Shannon index** | | | **Singletons** | | | **Doubletons** | | | **Coverage** | | |
| --- | --- | --- | --- | --- | --- | --- | --- | --- | --- | --- | --- | --- | --- | --- | --- | --- | --- | --- | --- |
| P1 | Wet | 339.2 | ± | 22.5 | 910.084 | ± | 57.11 | 3.948 | ± | 0.21 | 237.4 | ± | 16.8 | 48.8 | ± | 3.7 | 0.91 | ± | 0.0 |
| P2 |  | 307.65 | ± | 31.2 | 633.591 | ± | 75.35 | 4.262 | ± | 0.15 | 185.9 | ± | 21 | 52.5 | ± | 4.9 | 0.93 | ± | 0.0 |
| P3 |  | 239.75 | ± | 37 | 510.443 | ± | 67.05 | 3.377 | ± | 0.44 | 146.5 | ± | 17.7 | 38.9 | ± | 5 | 0.95 | ± | 0.0 |
|  |  |  |  |  |  |  |  |  |  |  |  |  |  |  |  |  |  |  |  |
| P1 | Dry | 205.7 | ± | 12.3 | 588.732 | ± | 13.66 | 3.267 | ± | 0.05 | 143.8 | ± | 8.3 | 26.9 | ± | 3 | 0.95 | ± | 0.0 |
| P2 |  | 229.95 | ± | 27.8 | 465.003 | ± | 64.66 | 3.828 | ± | 0.23 | 136.4 | ± | 17.4 | 39.2 | ± | 4.1 | 0.95 | ± | 0.0 |
| P3 |  | 348.2 | ± | 33.7 | 684.439 | ± | 31.14 | 4.198 | ± | 0.36 | 203.2 | ± | 13 | 61.2 | ± | 8.1 | 0.92 | ± | 0.0 |
